# Conserved Fever Pathways across Vertebrates: A Herpesvirus Expressed Decoy TNF-α Receptor Delays Behavioral Fever in Fish

**DOI:** 10.1016/j.chom.2017.01.010

**Published:** 2017-02-08

**Authors:** Krzysztof Rakus, Maygane Ronsmans, Maria Forlenza, Maxime Boutier, M. Carla Piazzon, Joanna Jazowiecka-Rakus, Derek Gatherer, Alekos Athanasiadis, Frédéric Farnir, Andrew J. Davison, Pierre Boudinot, Thomas Michiels, Geert F. Wiegertjes, Alain Vanderplasschen

**Affiliations:** 1 Immunology-Vaccinology, FARAH, Faculty of Veterinary Medicine, University of Liège, Liège 4000, Belgium; 2 Department of Evolutionary Immunology, Institute of Zoology, Jagiellonian University, Krakow 30-387, Poland; 3 Cell Biology and Immunology Group, Department of Animal Sciences, Wageningen University and Research, Wageningen 6708WD, the Netherlands; 4 Maria Sklodowska-Curie Memorial Cancer Center and Institute of Oncology, Gliwice 44-101, Poland; 5 Division of Biomedical and Life Sciences, Faculty of Health and Medicine, Lancaster University, Lancaster LA1 4YW, UK; 6 Instituto Gulbenkian de Ciência, Oeiras 2781-156, Portugal; 7 Biostatistics and Bioinformatics, FARAH, University of Liège, Liège 4000, Belgium; 8 MRC - University of Glasgow Centre for Virus Research, Glasgow G61 1QH, UK; 9 Virologie et Immunologie Moléculaires, INRA, Jouy-en-Josas 78352, France; 10 de Duve Institute, Université Catholique de Louvain, Brussels 1200, Belgium

**Keywords:** behavioral fever, fever, herpesvirus, cyprinid herpesvirus 3, host-virus interactions, pathogen-host-environment interplay, immune evasion mechanisms, innate immunity, tumor necrosis factor α, viral decoy receptor for cytokine

## Abstract

Both endotherms and ectotherms (e.g., fish) increase their body temperature to limit pathogen infection. Ectotherms do so by moving to warmer places, hence the term “behavioral fever.” We studied the manifestation of behavioral fever in the common carp infected by cyprinid herpesvirus 3, a native carp pathogen. Carp maintained at 24°C died from the infection, whereas those housed in multi-chamber tanks encompassing a 24°C–32°C gradient migrated transiently to the warmest compartment and survived as a consequence. Behavioral fever manifested only at advanced stages of infection. Consistent with this, expression of CyHV-3 ORF12, encoding a soluble decoy receptor for TNF-α, delayed the manifestation of behavioral fever and promoted CyHV-3 replication in the context of a temperature gradient. Injection of anti-TNF-α neutralizing antibodies suppressed behavioral fever, and decreased fish survival in response to infection. This study provides a unique example of how viruses have evolved to alter host behavior to increase fitness.

## Highlights

• Behavioral fever exhibited by carp in response to CyHV-3 infection is host beneficial
• CyHV-3 ORF12 delays behavioral fever expression, thereby promoting its own replication
• CyHV-3 ORF12 encodes a soluble decoy receptor for TNF-α
• TNF-α is a mediator of behavioral fever expressed by CyHV-3 infected carp

## Graphical Abstract

**Figure:**
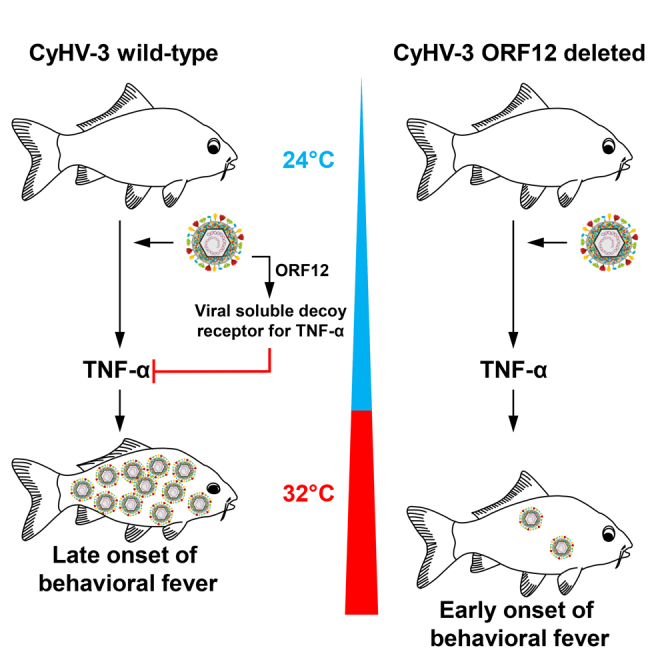

## Teaser

Ectotherms can express behavioral fever to limit pathogen infection. Rakus et al. show that a carp herpesvirus delays behavioral fever by expressing a soluble decoy receptor for TNF-α, thus promoting its own replication. In addition, they demonstrate a role for TNF-α in the induction of behavioral fever in teleost fish.

## Introduction

When infected by pathogens, endotherms and ectotherms can both increase their body temperature to limit the infection. In endotherms, this cardinal response to infection is called fever (for a recent review, see Evans et al., 2015). It relies mainly on thermogenesis, and also on physiological and behavioral modifications leading to reduced heat loss by the body. With the exception of a few rare examples, ectotherms lack intrinsic thermogenesis and so have a body temperature very close to the temperature of the environment. In a temperature gradient, ectotherms select a species-specific range of preferred temperature, which is defined as final thermal preferendum (FTP) (for a recent review, see Rakus et al., 2017). In response to infection or injection of exogenous pyrogens, ectotherms can increase their body temperature above their FTP through migration to warmer environments. This phenomenon is known as behavioral fever and is defined as an acute increase of the FTP consecutive to an infection (Rakus et al., 2017). Behavioral fever has been reported in a broad range of ectotherms including vertebrates (fishes, amphibians, and reptiles) and invertebrates.

Regulation of behavioral fever in ectotherms is evolutionarily related to fever in endotherms at various levels of the relevant regulatory pathways (Evans et al., 2015, Rakus et al., 2017), including the roles of exogenous pyrogens as inducers, the importance of the hypothalamic preoptic area as an integration site for pyrogenic signals, and the key role of prostaglandins as effector mediators. However, no study has yet determined whether this evolutionary relationship extends to the endogenous pyrogens, namely the cytokine mediators that inform the brain of exogenous pyrogens detected by immune cells throughout the body. In endotherms, cytokines such as interleukin 1β (IL-1β), IL6, tumor necrosis factor α (TNF-α), and interferons (Dinarello, 1999, Netea et al., 2000) have been shown to act as endogenous pyrogens.

At least in some infectious models, fever in endotherms and behavioral fever in ectotherms can increase host survival (Evans et al., 2015, Rakus et al., 2017). This beneficial effect is the consequence of the elevation of body temperature, which enhances the efficiency of both innate and (when existing) adaptive immune mechanisms and can restrict replication of invading pathogens. Through the expression of dedicated genes, pathogens are able to manipulate virtually all the physiological processes of their host that can affect their replication and transmission. However, to date, there is no report of a pathogen being able to affect the expression of behavioral fever by its host.

Cyprinid herpesvirus 3 (CyHV-3) is the causative agent of a lethal, highly contagious and notifiable disease in common and koi carp (*Cyprinus carpio*) (Boutier et al., 2015a). The outcome of CyHV-3 infection is highly dependent on temperature both in vitro and in vivo, with temperatures between 18°C and 28°C allowing viral replication in vitro and development of CyHV-3 disease in vivo, whereas temperatures above 30°C rapidly block CyHV-3 replication and the development of CyHV-3 disease (Gilad et al., 2003). During our studies of CyHV-3 pathogenesis (Boutier et al., 2015b), we observed that carp infected at 24°C (within the FPT of healthy carp) tended to concentrate around the tank heater when it was running. This observation led us to hypothesize that infected subjects might express behavioral fever in natural environments where temperature gradients exist (Boehrer and Schultze, 2008).

Here, we used the infection of carp by CyHV-3 as a homologous virus-host model to study the expression of behavioral fever. We demonstrate the ability of this virus to alter this behavior of its host through the expression of a single gene and identify the role of TNF-α as a mediator of behavioral fever in ectotherms.

## Results

### Carp Express Behavioral Fever in Response to CyHV-3 Infection

**Figure 1:**
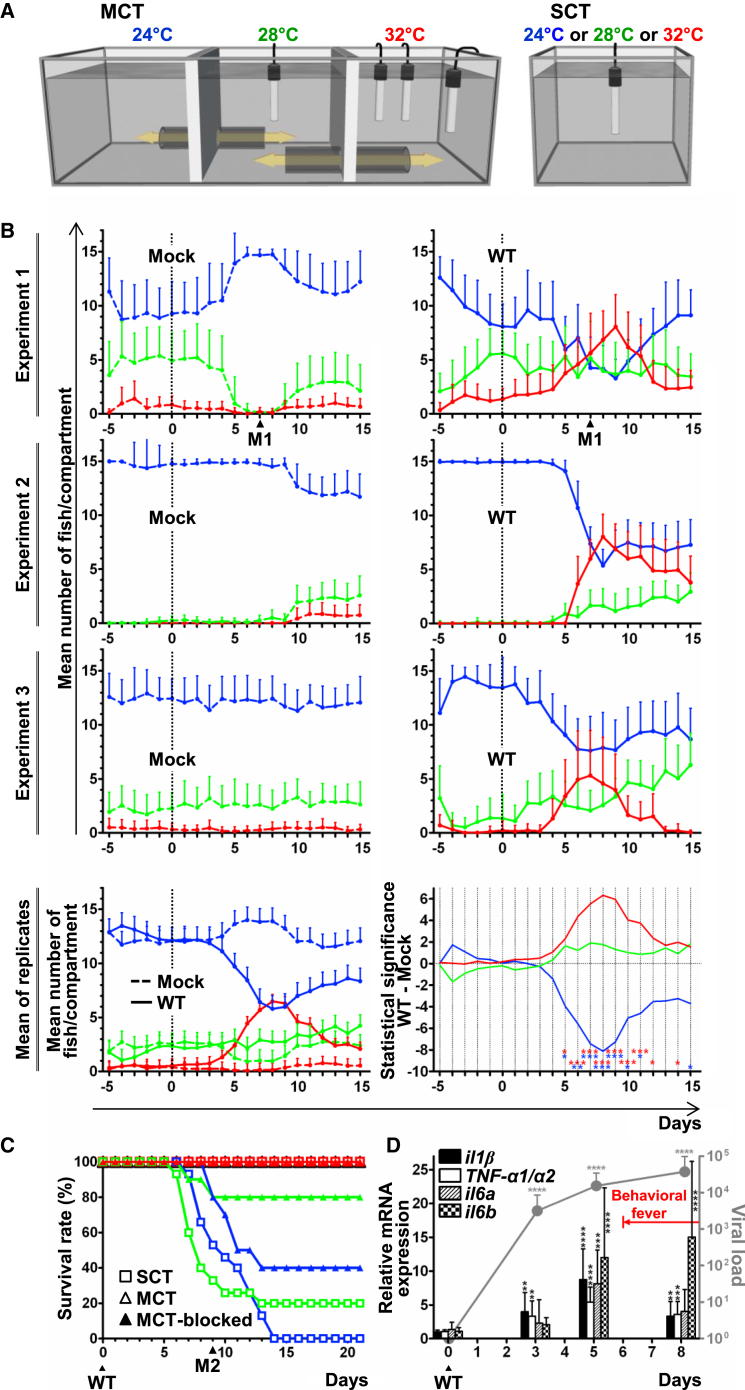
Carp Express Beneficial Behavioral Fever in Response to CyHV-3 Infection (A) Experimental setup tank systems used: multi-chamber tank (MCT) and single-chamber tank (SCT). Unless stated in the figure, in vivo experiments were performed in MCTs. In this and subsequent figures, a color code was adopted to illustrate temperature, with blue, green, and red representing 24°C, 28°C, and 32°C, respectively. See also Table S1. (B) Carp (n = 15 per tank) were housed in 6 MCTs. On day 0, fish were infected with wild-type CyHV-3 (WT) (right column, 3 upper graphs) or were mock-infected (Mock) (left column, 3 upper graphs). The number of fish in each compartment was counted every 30 min and expressed as a mean per day + SD. See also Movie S1 illustrating the first pair of MCTs at 7 dpi. The two lower graphs represent a global analysis of the data from the three replicates. The left graph presents the mean number + SD of fish in the different compartments of the MCTs based on the data of the three replicates. The statistical significance of the differences between the mean number of fish observed for WT infected and mock-infected groups is presented in the right graph. The days on which the number of fish per compartment was statistically different between WT infected and mock-infected fish are indicated according to the level of significance. (C) The effect of temperature on survival rate after CyHV-3 infection. Carp were housed in SCTs (n = 15), in a MCT (n = 15) (MCT) and in each compartment (n = 10) of a MCT in which the tunnels were blocked by grids (MCT-blocked). Survival rates were measured according to time post-infection with CyHV-3 (WT) (see also Movie S2 recorded 9 dpi for the MCT-blocked). (D) Carp (n = 15 per tank) were housed in 6 MCTs: 2 MCTs (1 mock-infected and 1 infected) were used for the observation of fish position, 4 MCTs were used for fish sampling (2 fish per time point per tank). At day 0, fish were mock-infected (1 MCT) or infected (5 MCTs) with CyHV-3 WT. At the indicated post-infection times, viral load (gray line) and cytokine gene expression were analyzed (n = 8; 2 fish were collected from each of the 4 replicate tanks leading to a total of 8 fish; mean + SD). Significant differences observed between CyHV-3 infected fish collected at different post-infection times and mock-infected fish are indicated by asterisks. The red arrow indicates the period during which expression of behavioral fever was significant (p < 0.05 or lower, significant increase of fish in the 32°C compartment).

First, we tested the hypothesis that common carp express behavioral fever in response to CyHV-3 infection. Carp were housed in multi-chamber tanks (MCTs; Figure 1A) where they could freely move between three compartments maintained at 24°C, 28°C, and 32°C. Fish distribution in the three compartments was recorded over time (Figure 1B). In the absence of infection (days −5 to 0), in all six observed MCTs, the majority of carp were distributed in the 24°C compartment and to a lesser extent in the 28°C compartment. At day 0, the fish in three MCTs were infected with CyHV-3, while the three remaining MCTs were mock-infected. Between 4 and 6 days post-infection (dpi), infected fish began to reside more frequently in the 32°C compartment. This observation is also illustrated in the supplemental file Movie S1, consisting of a short video shot at 7 dpi (MCTs of experiment 1). The number of infected fish in the 32°C compartment peaked at around 7–9 dpi. By 13 dpi, the distribution of fish returned to normal, with only the occasional fish in the 32°C compartment (Figure 1B). Global statistical analyses of the data from the three replicates demonstrated that carp infected by CyHV-3 express behavioral fever as revealed by a significantly higher number of fish in the 32°C compartment (p < 0.001) between 6 to 11 dpi. Interestingly, none of the fish infected with CyHV-3 in the MCTs died during the course of these experiments, suggesting that expression of behavioral fever could be beneficial for CyHV-3 infected carp.

### Expression of Behavioral Fever Is Beneficial for CyHV-3 Infected Carp

Carp were distributed in single chamber tanks (SCT; Figure 1A) maintained at 24°C, 28°C, or 32°C, or in the different compartments of a MCT in which the tunnels between the compartments were blocked by grids (MCT-blocked) or in a MCT. At time 0, all fish were infected with CyHV-3 (Figure 1C). Survival rates of 0% and 20% were recorded in SCTs maintained at 24°C and 28°C, respectively. Significantly higher survival rates of 40% (p = 0.033) and 80% (p = 0.004) were observed when infected fish were blocked by grids in the 24°C and 28°C compartments of the MCT, respectively. Consistent with an earlier report (Gilad et al., 2003), infected fish in the SCT at 32°C or blocked in the 32°C compartment of the MCT did not develop CyHV-3 disease. The effect of temperature on the development of CyHV-3 disease is also illustrated by Movie S2. It shows that clinical signs expressed by fish blocked in MCT compartments were inversely related to temperature. Importantly, all the fish infected in the MCTs survived the infection as the consequence of the expression of behavioral fever.

### Expression of Behavioral Fever Occurs at an Advanced Stage of CyHV-3 Disease

The data above demonstrate that expression of behavioral fever is beneficial for CyHV-3 infected carp. However, clinical observation of fish in the MCTs revealed that their migration to the warmest compartment occurred only at an advanced stage of the disease. To verify the relatively late onset of behavioral fever with respect to viral replication and cytokine upregulation, fish infected in MCTs were analyzed over time for viral load and carp proinflammatory cytokine gene expression. Data presented in Figure 1D confirmed that the onset of behavioral fever observed in this experiment at 6 dpi occurred days after systemic replication of the virus (significant at 3 dpi) and upregulation of proinflammatory cytokines (significant for *il1β* and *TNF-α1/α2* at 3 dpi and for all cytokines tested at 5 dpi).

The relatively late onset of behavioral fever led us to postulate that this phenomenon might be delayed by the virus in order to retain its host at a temperature compatible with viral replication. As some viruses have been shown to express soluble decoy cytokine receptors (Epperson et al., 2012), and as the CyHV-3 genome potentially encodes such receptors (Yi et al., 2015), we hypothesized that CyHV-3 might express decoy receptor(s) able to neutralize putative pyrogenic cytokines produced by fish. CyHV-3 ORF12 was selected as a candidate because it encodes a putative soluble TNF-receptor homolog that is the most abundant viral protein of the CyHV-3 secretome (Ouyang et al., 2013).

### CyHV-3 ORF12 Deletion Does Not Affect Viral Replication In Vitro or Virulence In Vivo under Standard Laboratory Conditions—SCT at 24°C

**Figure 2:**
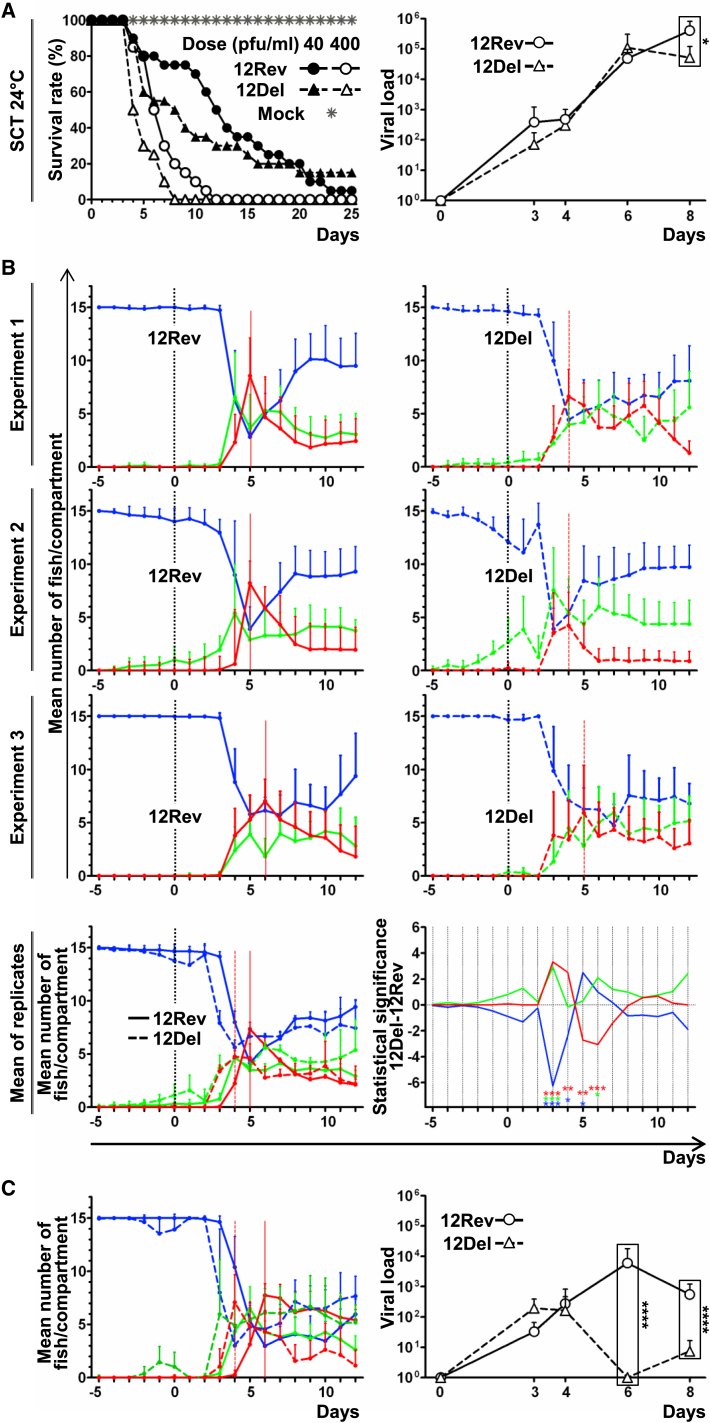
In Vivo Phenotyping of CyHV-3 ORF12 Deletion (A) Fish were housed in SCT at 24°C. At time 0, fish were infected with the 12Del or 12Rev versions of CyHV-3. Cumulative survival rates of carp (n = 20) mock-infected or infected with the indicated doses of CyHV-3 ORF12 recombinant strains were measured (left graph). The right graph presents the kinetics of viral load according to time post-infection with 12Rev or 12Del (dose of 100 pfu/mL). At the indicated times post-infection, viral load was measured in gills (a total of 6 fish originating from duplicate tanks [3 fish per tank] were analyzed per time point, mean + SD). For each time point, the results obtained for 12Rev and 12Del were analyzed for significant differences (marked by asterisks). (B) Carp (n = 15 per tank) were housed in 6 MCTs. On day 0, fish were infected with 12Rev (left column, 3 upper graphs) or 12Del (right column, 3 upper graphs). The number of fish in each compartment was expressed as a mean per day + SD. The two lower graphs represent the global analysis of the data from the three replicates as described in Figure 1B. The statistical significance of the differences between the mean number of fish observed for 12Del and 12Rev groups is presented in the right graph. (**C**) Carp (n = 15 per tank) were housed in 6 MCTs. On day 0, fish were infected with 12Rev (3 tanks) or 12Del (3 tanks). One randomly selected tank per viral genotype was selected for monitoring the position of the fish (left graph), and the two remaining tanks were used to analyze viral load according to time post-infection. At the indicated post-infection times, viral load was measured in gills (per time point, a total of 6 fish originating from the duplicate tanks [3 fish/tank], mean + SD). For each time point, the results obtained for 12Rev and 12Del were analyzed for significant differences (marked by asterisks).

To investigate whether CyHV-3 is able to delay the expression of behavioral fever through expression of ORF12, a CyHV-3 ORF12 deletion (12Del) mutant and a revertant (12Rev) virus (in which ORF12 was restored) were derived from the parental wild-type (WT) strain (Figure S1). The structure and transcription of the ORF12 region, as well as the full-length genome sequences, were validated in all viral strains (Supplemental Information and Figure S1). Next, we characterized the phenotype of ORF12 deletion in vitro and in vivo. In cell culture, the three strains replicated comparably at 24°C and 28°C (Figure S1E). As reported earlier, CyHV-3 did not replicate at 32°C (Gilad et al., 2003). Since the WT and the 12Rev viruses exhibited identical genome sequences, only the latter was used in in vivo experiments. Inoculation of fish in SCTs at 24°C did not reveal a phenotype for 12Del different from that of the 12Rev (Figure 2A). Thus, similar clinical signs (data not shown) and survival rates (Figure 2A; left graph) were observed for 12Del and 12Rev. Moreover, viral load in gills increased similarly for the two viruses (Figure 2A; right graph). These observations demonstrate that ORF12 deletion does not exhibit a phenotype different from that of the 12Rev under standard laboratory conditions.

### CyHV-3 ORF12 Delays the Expression of Behavioral Fever and Promotes CyHV-3 Replication in an Environment Encompassing a Temperature Gradient

In parallel to the experiments described above (Figure 2A), infections were also performed in MCTs (Figure 2B). Carp were distributed in 6 MCTs. At time 0, three MCTs were infected with 12Rev and the three others with 12Del. These triplicate experiments revealed that fish infected with 12Del expressed behavioral fever significantly earlier than those infected with 12Rev. Independently of the viral genotype, no fish died from CyHV-3 infection in MCTs, but because of their earlier migration to the warmest compartment, fish infected with 12Del expressed less severe clinical signs compared to fish infected with 12Rev. To verify this phenotypic difference between 12Del and 12Rev, which was not observed in SCTs at 24°C (Figure 2A), an independent infection with the two viruses was performed in 6 MCTs (3 MCTs per genotype), two intended for observation of fish distribution and four for sample collection, in order to measure viral load at various times post-infection (Figure 2C). In this experiment, fish infected with 12Del migrated 2 days earlier to the warmest compartment (Figure 2C; left graph). Interestingly, although the two viruses exhibited comparable replication kinetics in the SCTs (Figure 2A; right graph) and in the MCTs before expression of behavioral fever, they differed drastically once the fish migrated to the 32°C compartment (Figure 2C; right graph). Due to their earlier migration to a non-permissive temperature, fish infected with 12Del showed a faster and more drastic decrease of viral load than those infected with 12Rev. These results demonstrate that CyHV-3 is able to alter the expression of behavioral fever by its host via the expression of a single gene, thus favoring its replication.

### CyHV-3 ORF12 Encodes a Soluble Decoy Receptor for TNF-α

**Figure 3:**
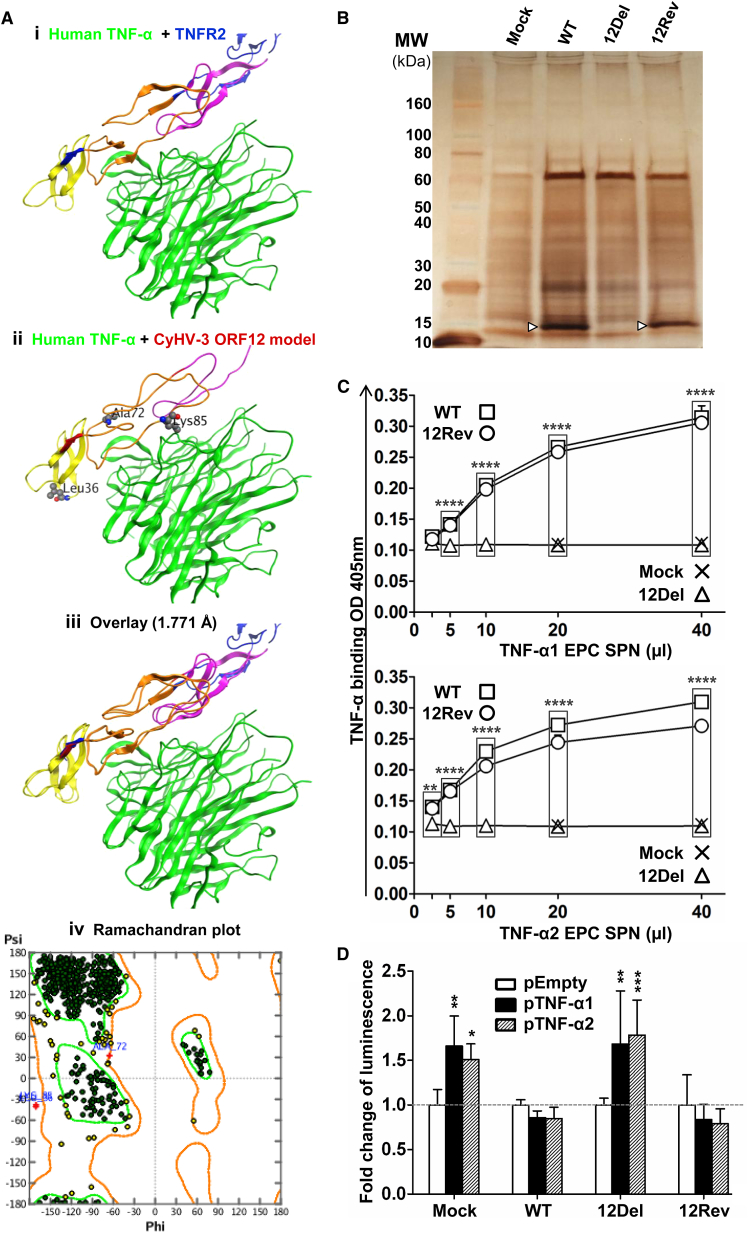
CyHV-3 ORF12 Encodes a Soluble Decoy Receptor for TNF-α (A) ORF12 homology modeling. (i) Human TNF-α (green) with human TNFR2 (c6 domains in yellow, orange, and pink, intervening sequences in blue; PDB: 3ALQ). (ii) Homology model (colored as in Ai) of CyHV-3 ORF12 on 3ALQ.R, with proposed interaction with TNF-α (green) also shown. Residues in ball-and-stick representation are those with poor bond angles as assessed by Ramachandran plot. (iii) Overlay of (i) and (ii), superposition at 1.771Å is achieved. (iv) Ramachandran plot. Bad angles and the position of the affected residues in (ii) are indicated in red. (B) Silver staining of total proteins found in concentrated supernatants of CCB cells infected with CyHV-3 (WT, 12Del, or 12Rev) or mock-infected (Mock). Arrowheads indicate the band corresponding to the ORF12 protein. (C) ELISA binding assay of carp TNF-α1 and TNF-α2 to CyHV-3 secreted proteins. The data are the mean + SD of duplicate measurements. Results for which a significant difference was observed between WT/12Rev and 12Del/Mock groups are marked by asterisks. See also Figure S2. (D) Neutralization of carp TNF-α1 and TNF-α2 by CyHV-3 secreted proteins was tested by using a TNF-α bioluminescent reporter assay. The data are expressed as fold change of luminescence relative to the respective controls and represent the mean + SD of quadruplicate measurements. Results for which a significant difference was observed between pTNF-α1 or pTNF-α2 and pEmpty are marked by asterisks. These data are representative of three independent experiments.

Next, we tried to unravel the mechanism by which ORF12 delays expression of behavioral fever. According to our initial hypothesis, ORF12 might act as a soluble decoy receptor that neutralizes pyrogenic cytokines. As ORF12 encodes a putative soluble Tnf-receptor homolog, and as TNF-α is an endogenous pyrogen in endotherms (Dinarello et al., 1986), we investigated whether ORF12 encodes a soluble decoy receptor for TNF-α. The amino acid sequence of the ORF12 protein was aligned manually onto the human TNFR2 protein by using the Pfam TNFR_c6 domains (Finn et al., 2014) as a guide. TNFR2 has three TNFR_c6 domains in tandem and ORF12 has two, plus a fragment of a third. The solved structure of the human TNF-TNFR2 complex (3ALQ; Figure 3Ai) (Mukai et al., 2010) was used to develop a homology model of CyHV-3 ORF12 (Figure 3Aii). Superposition was achieved at 1.771Å (Figure 3Aiii) and favorably assessed by Ramachandran plot (Figure 3Aiv). The quality of the homology model strongly suggested that ORF12 forms a structure that contains at least two functional TNFR_c6 domains and that could bind TNF-α. The top BLASTP hit between ORF12 and eukaryotic proteins is the TNFR-2A from *Onchorhyncus mykiss* at 48% identity. The top hits with herpesvirus sequences are also in this range: UL144 in chimpanzee cytomegalovirus at 42% and ORF150D in cyprinid herpesvirus 1 at 40%.

To test the hypothesis that ORF12 encodes a functional soluble TNF-α receptor, concentrated supernatants were produced from cell cultures infected with CyHV-3 WT, 12Del, and 12Rev, as well as from mock-infected cultures. Silver staining of total supernatant proteins confirmed that ORF12 (predicted molecular mass 12.6 kDa) is the most abundant viral protein in the CyHV-3 secretome (Figure 3B). To test the ability of ORF12 to bind carp TNF-α, proteins of concentrated supernatants were coated on ELISA plates before incubation with carp TNF-α1 or TNF-α2 (Figure 3C). Carp TNF-α1 and TNF-α2 are both homologs of mammalian TNFSF2 (TNF-α) (Forlenza et al., 2009). Quantification of TNF-α binding demonstrated that the WT and 12Rev supernatants contained TNF-α-binding activity, in contrast to the 12Del and mock-infected supernatants (Figure 3C). These results were confirmed by independent ELISA combining a different detection system (anti-His-tag monoclonal antibody) and an alternative prokaryotic source of TNF-α1/ TNF-α2 (Figure S2). Next, a bioluminescence reporter assay was used to determine whether ORF12 binding to TNF-α could neutralize its ability to activate NF-κB signaling (Figure 3D). When incubated with concentrated supernatants from mock- or 12Del-infected cells, carp TNF-α1 and TNF-α2 induced activation of NF-κB similarly. This activation was completely inhibited when the cytokines were pre-incubated with WT or 12Rev supernatants (Figure 3D). These data demonstrated that ORF12 encodes a soluble TNF-α receptor able to neutralize both TNF-α1 and TNF-α2 from carp.

### TNF-α Is a Mediator of Behavioral Fever Expressed by CyHV-3 Infected Carp

**Figure 4:**
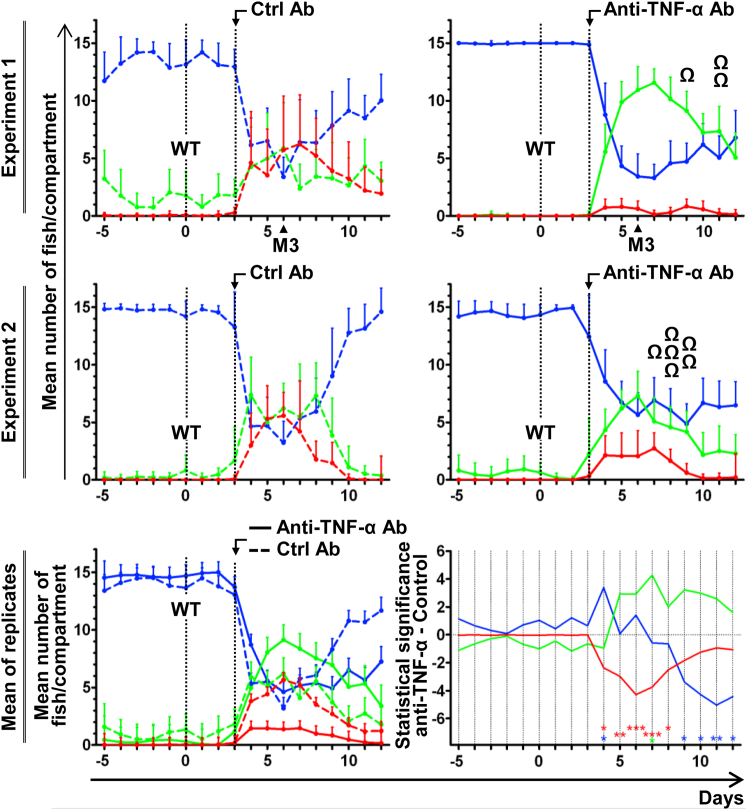
Anti-TNF-α Antibodies Inhibit the Expression of Behavioral Fever Carp (n = 15 per tank) were housed in 4 MCTs. On day 0, fish were infected with wild-type CyHV-3 (WT) and then injected 3 days later with control (Ctrl) (left column, 2 upper graphs) or anti-TNF-α (right column, 2 upper graphs) antibodies. Distribution of fish in the MCT compartments was analyzed and expressed as a mean per day + SD. The Ω symbol illustrates fish that died from the infection. See also Movie S3 recorded 6 dpi for the first replicate. The two lower graphs represent the global analysis of the data from the two replicates as described in Figure 2B. The days at which the number of fish per compartment was statistically different between the control and anti-TNF-α groups are indicated according to the level of significance.

The ability of CyHV-3 ORF12 to delay the expression of behavioral fever, together with the results above showing that ORF12 encodes a decoy receptor able to neutralize TNF-α, led us to hypothesize that TNF-α might act as a pyrogenic cytokine in ectotherms, as it does in endotherms. To explore this hypothesis, carp were distributed in 4 MCTs and infected at time 0 with the CyHV-3 WT strain and then, 3 days later, injected intraperitoneally with control irrelevant or anti-TNF-α neutralizing antibodies (2 MCTs per condition; Figure 4). Injection of anti-TNF-α neutralizing antibodies induced a significant reduction of migration to the 32°C compartment, and as a consequence only 80% and 60% of the fish survived. By contrast, infected fish injected with irrelevant antibodies migrated efficiently to the 32°C compartment, and all survived the infection. This observation is also illustrated by Movie S3 recorded 6 dpi for the first replicate. Injection of anti-TNF-α neutralizing antibodies did not reduce migration of fish to the 28°C compartment, suggesting the partial in vivo neutralization of TNF-α or the implication of other pyrogenic cytokines. However, these results demonstrate that carp TNF-α is a mediator of behavioral fever in our model.

## Discussion

Here, we studied the expression of behavioral fever by using a relevant biological model in which CyHV-3 infects its natural host through a natural route. Our data highlight the importance of the environment in the pathogen-host-environment interplay. Importantly, they also demonstrate the ability of a virus to alter the behavior of its vertebrate host through the expression of a single gene and identify the role of TNF-α as a mediator of behavioral fever in ectotherms.

Depending on the environment, CyHV-3 infection can generate extreme mortality rates. In environments that allow the expression of behavioral fever at 32°C, no mortality was observed (Figure 1C). In contrast, in some conditions in which this behavior cannot be expressed, mortality reached 100%. These observations suggest that the virulence of pathogens that infect ectotherms and are inhibited by behavioral fever can be exacerbated by environmental changes that prevent their host from expressing this innate immune response.

The present study demonstrates a beneficial effect of behavioral fever in response to a specific pathogen in a specific host. However, challenge trials using different fish species and different pathogens revealed that an increase of temperature correlated with an increase in mortality rate (exemplified by Jun et al., 2009), suggesting that expression of behavioral fever in these models would be maladaptive. It will be interesting in the future to determine whether fish can express a response against some pathogens opposite to behavioral fever (i.e., seeking cooler water) and whether this can confer an advantage to the infected subject.

Viruses are able to manipulate virtually all the physiological processes of their host to enhance their replication and transmission. However, there are very few reports of viruses that alter host behavior in a way that might increase their fitness. A frequently cited example is rabies virus, which, through the neurological lesions it causes, induces an aggressive behavior favoring the contamination of naive subjects by infectious saliva (John et al., 2015). To the best of our knowledge, there are only two reports of viruses able to alter the behavior of their host through the effect of identified genes, and both are related to baculoviruses, which have been shown to increase the locomotor activity and climbing behaviors of infected caterpillar hosts (Kamita et al., 2005, Hoover et al., 2011). To date, no pathogen has been shown to alter the expression of behavioral fever. We have demonstrated that CyHV-3 encodes a gene that delays the expression of behavioral fever, thereby enhancing viral replication in a viral excretion organ. Two non-exclusive hypotheses could explain the selective advantage conferred by the ability to delay the expression of behavioral fever. First, it could enhance viral replication and excretion by retaining infected fish at a permissive temperature. Second, it could favor viral transmission by retaining infected fish at the temperature preferred by non-infected fish, thereby promoting physical contact between them. It is notable that ORF12 delays the behavioral fever response rather than completely inhibiting it. This time-dependent effect likely reflects a selective advantage for CyHV-3. As indicated above, by delaying the behavioral fever response, ORF12 is likely to enhance viral spread through a fish population. Furthermore, by allowing fish finally to express behavioral fever, and thus survive the infection, the virus could promote the appearance of latently infected fish that will carry and spread the herpesvirus infection throughout their lives (Reed et al., 2014). Experiments are in progress to test these hypotheses.

Here, we identified the role of carp TNF-α as a key mediator of behavioral fever in an ectotherm (Figure 4). Our results suggest that behavioral fever in ectotherms and fever in endotherms are evolutionarily and functionally related through common cytokine mediators that originated more than 400 million years ago. The results of this study suggest that the ancestral signaling pathway of behavioral fever regulation in ectotherms also evolved in endotherms to regulate the expression of fever. Finally, our results support the importance of the interplay between the immune system and the central nervous system.

In conclusion, this study demonstrates the ability of a vertebrate virus to alter the behavior of its host through the expression of a single gene. This gene encodes a soluble viroreceptor able to bind and neutralize TNF-α, which was shown to be a mediator of behavioral fever in our homologous model. An interesting perspective would be to investigate whether the homology existing between the cytokine signaling pathways of behavioral fever in ectotherms and fever in endotherms extends to cytokines other than TNF-α.

## Experimental Procedures

### Cells

*Epithelioma papulosum cyprini* (EPC) and *Cyprinus carpio* brain (CCB) cells were cultured as described previously (Boutier et al., 2015b, Forlenza et al., 2009).

### TNF-α Eukaryotic Expression Vectors and Anti-TNF-α Antibodies

Plasmids pIRES-TNF-α1-EGFP and pIRES-TNF-α2-EGFP (hereafter referred to as pTNF-α1 and pTNF-α2, respectively) (Forlenza et al., 2009) were used. The empty vector, pIRES-EGFP (hereafter referred to as pEmpty), was used as a negative control. Affinity-purified polyclonal rabbit anti-carp TNF-α IgG neutralizing both carp TNF-α1 and TNF-α2 (anti-TNF-α) (Forlenza et al., 2009), as well as purified polyclonal rabbit irrelevant control IgG (CT), were used.

### Production and Characterization of CyHV-3 ORF12 Recombinant Strains

A CyHV-3 ORF12 deletion (12Del) mutant and a revertant (12Rev) virus (in which ORF12 was restored) were derived from the parental, wild-type (WT) FL BAC clone (Figures S1A and S1B) by using BAC cloning and prokaryotic recombination technologies (Boutier et al., 2015b). The structure and transcription of the ORF12 region, as well as the full-length genome sequences, were validated in all viral strains (Supplemental Information and Figure S1). The ability of the recombinants to replicate in cell culture was investigated by multi-step growth curves as described previously (Boutier et al., 2015b) (Figure S1E). A more detailed version of this section is provided in the Supplemental Experimental Procedures.

### Concentrated Cell Supernatants

Cultures of CCB cells were infected with CyHV-3 at a multiplicity of infection (MOI) of 0.1 plaque-forming units (pfu)/cell or mock-infected. Concentrated cell supernatants were produced as described previously (Ouyang et al., 2013).

### TNF-α Binding Assay

Binding of carp TNF-α to the CyHV-3 secretome was analyzed by ELISA (Forlenza et al., 2009). A detailed description of this method is available in the Supplemental Experimental Procedures.

### TNF-α Bioluminescent Reporter Assay

EPC cells stably transfected with pNiFty2-Luc (InvivoGen) (Piazzon et al., 2015), referred to as EPC-NFκB-Luc cells, were used to measure TNF-α bioactivity by bioluminescence (see the Supplemental Experimental Procedures for a detailed description of this method).

### Ethics Statement

The animal studies were approved by the local ethics committee (Laboratory accreditation No. 1610008, protocol No. 1327).

### Fish

European common carp (*Cyprinus carpio carpio*) between 7 and 11 months old and weighing between 8 and 12 g were used. Experimental replicates were performed with contemporary offspring derived from one female and one male carp.

### Tank Systems

A single-chamber tank (SCT) system and a multi-chamber tank (MCT) system were used (Figure 1A; see also Movie S1 and Supplemental Experimental Procedures). Temperatures in all three compartments of MCTs were controlled by measurements every 30 min. The observed temperatures of the MCTs used for the experiments in this manuscript are presented as the mean ± SD in Table S1.

### Inoculation and Injection of Fish

For inoculation with CyHV-3, fish were immersed for 2 hr in water containing the virus at a dose of 100 pfu/mL, unless otherwise specified. Antibodies (1 μg/g of fish body weight) were injected intraperitoneally.

### Monitoring of Fish Position in the MCTs

The number of fish in each compartment was counted manually from the images captured by a digital camera at each successive 30 min, resulting in 48 measurements per day. The results are presented as the mean + SD (n = 48) of the number of fish observed per day in each compartment (for additional information see Supplemental Experimental Procedures).

### Sampling of Fish Tissues

Fish were euthanized by immersion in water containing benzocaine (100 mg/L). Tissue samples were collected immediately, placed in RNAlater (Invitrogen), and stored at −80°C.

### Quantification of Viral Genome Copies by qPCR

The viral genome (viral genome copies/10^6^ carp *glucokinase* gene copies (log10)) was quantified by real-time TaqMan qPCR as described previously (Boutier et al., 2015b). The primers and probes are listed in the Supplemental Experimental Procedures.

### Quantification of Carp Gene Expression by qRT-PCR

The expression of target genes was measured by qRT-PCR as described previously (Ouyang et al., 2013, Rakus et al., 2012). The primers used are listed in the Supplemental Experimental Procedures.

### Homology Modeling

Homology models for ORF12 were constructed against 3ALQ using MOE 2015.10 (Chemical Computing Group, Montreal). The datasets for the homology models for CyHV-3 ORF12 reported in this paper are available at the Lancaster University database: http://dx.doi.org/10.17635/lancaster/researchdata/28.

### Statistical Analyses

All analyses were performed by using SAS (v9.3). A detailed description of the statistical analyses used is available in the Supplemental Experimental Procedures. For all analyses, significance is set at the 0.05 threshold (−, not significant; ^∗^p < 0.05; ^∗∗^p < 0.01; ^∗∗∗^p < 0.001; ^∗∗∗∗^p < 0.0001).

## Author Contributions

K.R., M.R., and A.V. conceived the study, performed most of the experiments, and wrote the manuscript. A.V. made the preliminary observation at the origin of this project and obtained funding for its completion. K.R. built the MCT and demonstrated the expression of behavioral fever. M.B. and J.J.-R. contributed to the experiments presented in Figures S1, S2, and 2, respectively. M.F., M.C.P., and G.F.W. designed and performed the experiments presented in Figures S2, 3C, and 3D. They also provided the antibodies used in Figure 4. A.A. and D.G. performed the in silico analyses (Figure 3A). F.F. performed statistical analyses. T.M. designed the experiments presented in Figures 2A and 2C. A.J.D. determined the genome sequences of CyHV-3 recombinants. P.B. contributed to the conceptualization of the study.

## Supplemental Information

Document S1. Supplemental Experimental Procedures, Figures S1 and S2, and Table S1

Movie S1. Common Carp Express Behavioral Fever in Response to CyHV-3 Infection, Related to Figure 1B This movie starts with an animated cartoon illustrating the structure of the MCT used in this study and how the MCTs were positioned in the room along a central axis of symmetry (warmest compartments being the closest to the center of the room). The video then presents movies of fish that were mock-infected (left and first presented MCT) or infected (right and second presented MCT) 7 days earlier with wild-type CyHV-3.

Movie S2. Effect of Water Temperature on the Development of CyHV-3 Disease, Related to Figure 1C This video starts with an animated cartoon illustrating the structure of a MCT in which the tunnels were blocked by grids. The video then presents a movie of fish that were infected 9 days earlier with wild-type CyHV-3 before their distribution into the compartments of the MCT.

Movie S3. Anti-TNF-α Antibodies Inhibit the Expression of Behavioral Fever Induced by CyHV-3 Infection, Related to Figure 4 This video starts with an animated cartoon explaining the flow chart of this experiment. Fish were first infected with CyHV-3 and then injected 3 days later with anti-TNF-α antibodies (left and first presented MCT) or irrelevant control antibodies (right and second presented MCT). Movies of the fish were recorded at 6 dpi.

Document S2. Article plus Supplemental Information
