# Supplementary material for: Conserved Fever Pathways across Vertebrates: A Herpesvirus Expressed Decoy TNF-α Receptor Delays Behavioral Fever in Fish
Source: Cell Host Microbe. 2017 Feb 8;21(2):244–53. doi: 10.1016/j.chom.2017.01.010 (PMC5301049; doi:10.1016/j.chom.2017.01.010)
Supplement: Document S1. Supplemental Experimental Procedures, Figures S1 and S2, and Table S1 [file mmc1.pdf]

Cell Host & Microbe, Volume *21*

Supplemental Information

Conserved Fever Pathways across Vertebrates: A Herpesvirus Expressed Decoy TNF-α Receptor Delays Behavioral Fever in Fish

Krzysztof Rakus, Maygane Ronsmans, Maria Forlenza, Maxime Boutier, M. Carla Piazzon, Joanna Jazowiecka-Rakus, Derek Gatherer, Alekos Athanasiadis, Frédéric Farnir, Andrew J. Davison, Pierre Boudinot, Thomas Michiels, Geert F. Wiegertjes, and Alain Vanderplasschen

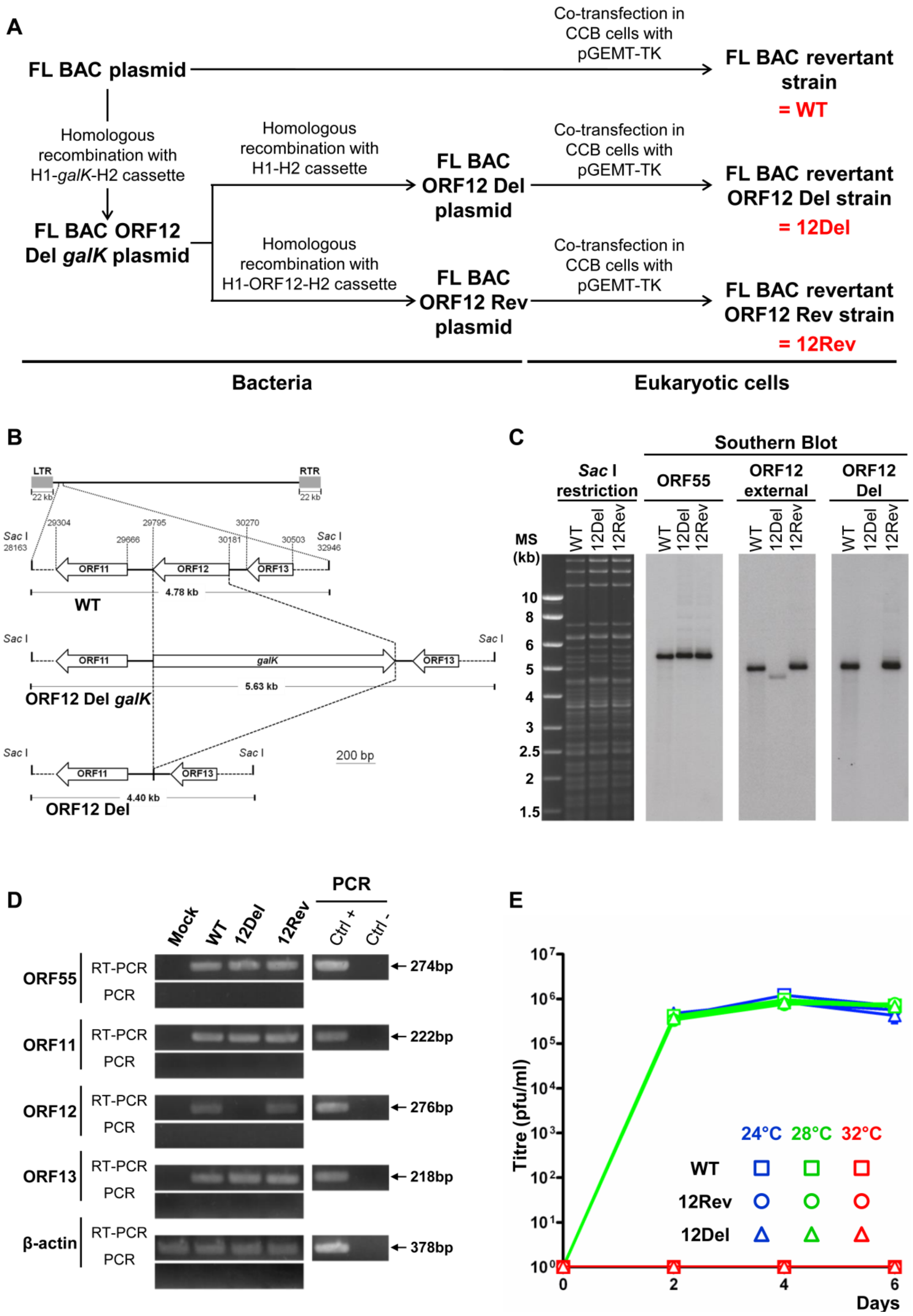
A
FL BAC plasmid
Co-transfection in CCB cells with pGEMT-TK
FL BAC revertant strain
= WT
Homologous recombination with H1-galK-H2 cassette
FL BAC ORF12 Del galK plasmid
Homologous recombination with H1-H2 cassette
FL BAC ORF12 Del plasmid
FL BAC revertant ORF12 Del strain
= 12Del
Homologous recombination with H1-ORF12-H2 cassette
FL BAC ORF12 Rev plasmid
FL BAC revertant ORF12 Rev strain
= 12Rev
Bacteria
Eukaryotic cells
B
LTR
RTR
22 kb
Sac I
ORF11
ORF12
ORF13
4.78 kb
WT
galK
5.63 kb
ORF12 Del galK
4.40 kb
ORF12 Del
200 bp
C
Southern Blot
Sac I restriction
ORF55
ORF12 external
ORF12 Del
MS (kb)
WT
12Del
12Rev
D
PCR
Mock
WT
12Del
12Rev
Ctrl +
Ctrl -
ORF55
ORF11
ORF12
ORF13
β-actin
RT-PCR
PCR
274bp
222bp
276bp
218bp
378bp
E
Titre (pfu/ml)
Days
24°C
28°C
32°C
WT
12Rev
12Del

Figure S1 related to Figure 2 – Production and characterization of CyHV-3 ORF12 recombinant strains. (**A**) Flow chart of steps performed to produce ORF12 recombinant plasmids and to reconstitute viral strains. (**B**) The region of the CyHV-3 genome encoding ORF12 is illustrated for wild type (WT), ORF12 Del *galK* and ORF12 Del genotypes. All coordinates correspond to the reference CyHV-3 sequence (NC_009127.1). (**C**) Structural analysis of the genome of ORF12 recombinant strains by *Sac* I restriction and Southern blotting. (**D**) RT-PCR analysis of the ORF12 genome region. (**E**) Effect of ORF12 deletion on viral growth *in vitro*. Replication kinetics of CyHV-3 ORF12 recombinant strains were compared with those of the WT strain using a multi-step growth assay at three different temperatures. The data presented are the means + SD of triplicate measurements.

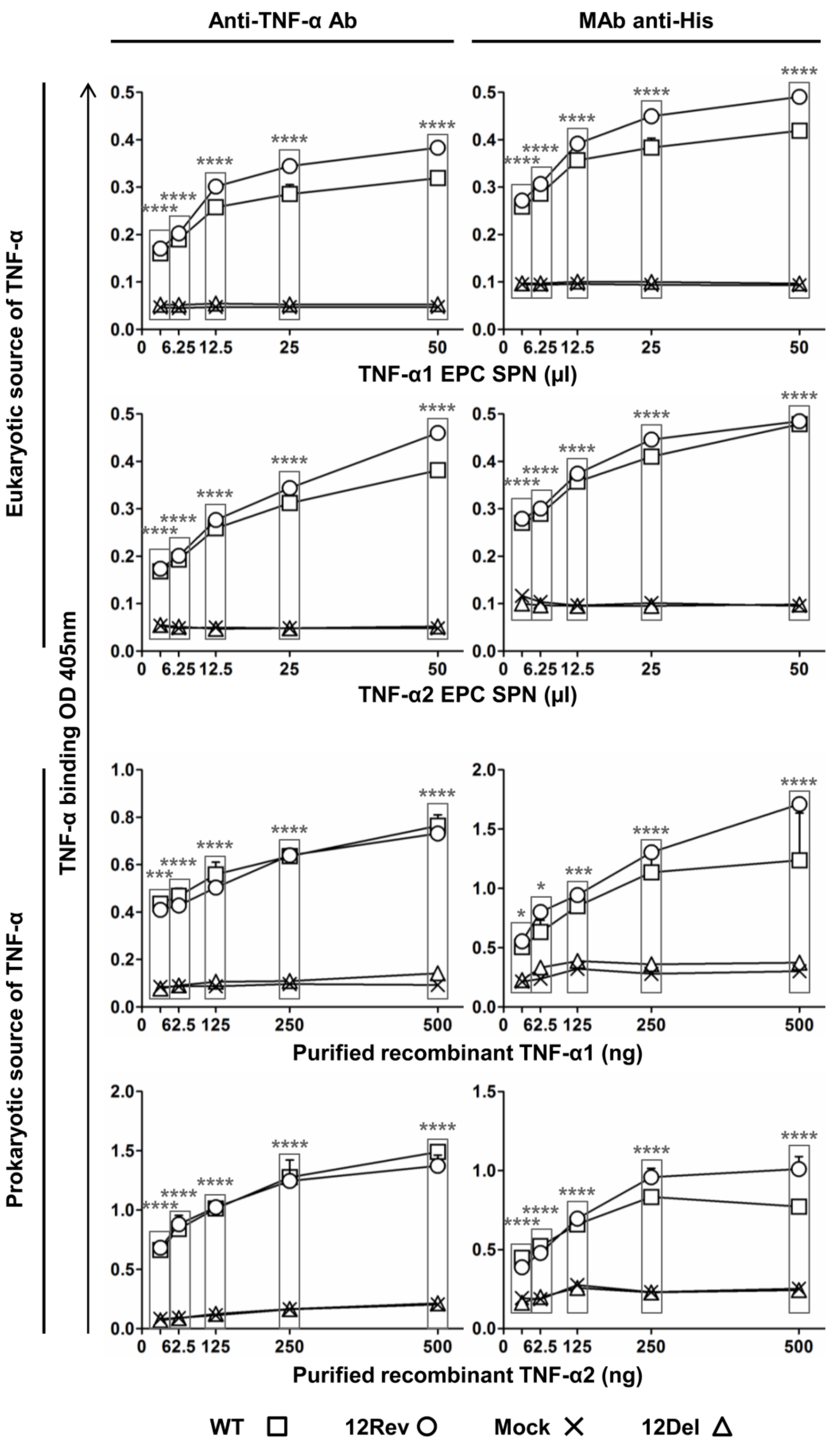

Anti-TNF-α Ab
MAb anti-His
Eukaryotic source of TNF-α
Prokaryotic source of TNF-α
TNF-α binding OD 405nm
TNF-α1 EPC SPN (µl)
TNF-α2 EPC SPN (µl)
Purified recombinant TNF-α1 (ng)
Purified recombinant TNF-α2 (ng)
WT
12Rev
Mock
12Del

Figure S2 related to Figure 3C. Binding of carp TNF-α1 and TNF-α2 to CyHV-3 secreted proteins. ELISAs were performed with two sources of carp TNF-α: supernatant of transfected EPC cells (upper half of the figure) and purified TNF-α1/ TNF-α2 expressed in bacteria (lower half). Binding of TNF-α1/ TNF-α2 was detected with polyclonal anti-TNF-α antibodies (left column) or with a MAb anti-His (right column). The data are the mean + SD of duplicate measurements. Results for which a significant difference was observed between the WT/12Rev and 12Del/Mock groups are marked by asterisks.

Movie S1 (M1) related to Figure 1B. Common carp express behavioral fever in response to CyHV-3 infection. This movie starts with an animated cartoon illustrating the structure of the MCT used in this study and how the MCTs were positioned in the room along a central axis of symmetry (warmest compartments being the closest to the center of the room). The video then presents movies of fish that were mock-infected (left and first presented MCT) or infected (right and second presented MCT) 7 days earlier with wild-type CyHV-3.

Movie S2 (M2) related to Figure 1C. Effect of water temperature on the development of CyHV-3 disease. This video starts with an animated cartoon illustrating the structure of a MCT in which the tunnels were blocked by grids. The video then presents a movie of fish that were infected 9 days earlier with wild-type CyHV-3 before their distribution into the compartments of the MCT.

Movie S3 (M3) related to Figure 4. Anti-TNF-α antibodies inhibit the expression of behavioral fever induced by CyHV-3 infection. This video starts with an animated cartoon explaining the flow chart of this experiment. Fish were first infected with CyHV-3 and then injected three days later with anti-TNF-α antibodies (left and first presented MCT) or irrelevant control antibodies (right and second presented MCT). Movies of the fish were recorded at 6 dpi.

Table S1. Temperature recorded in the compartments of MCTs (Related to Experimental Procedures (tank systems) and to the Figures listed in the first column)

Figure	Experiment	Tank	Virus/antibody	24°C compartment	28°C compartment	32°C compartment
**Fig. 1B**	**Experiment 1**	MCT	Mock	23.4 ± 0.57°C	27.9 ± 0.41°C	33.9 ± 0.42°C
		MCT	WT	22.6 ± 0.78°C	27.7 ± 0.49°C	33.9 ± 0.55°C
**Fig. 1B**	**Experiment 2**	MCT	Mock	24.8 ± 0.20°C	28.1 ± 0.14°C	32.4 ± 0.07°C
		MCT	WT	23.8 ± 0.25°C	26.8 ± 0.26°C	30.7 ± 0.51°C
**Fig. 1B**	**Experiment 3**	MCT	Mock	24.6 ± 0.23°C	29.0 ± 0.05°C	32.1 ± 0.39°C
		MCT	WT	24.9 ± 0.88°C	27.9 ± 0.80°C	31.6 ± 0.92°C
**Fig. 1C**		MCT	WT	24.6 ± 0.40°C	28.1 ± 0.14°C	32.2 ± 0.25°C
		MCT blocked	WT	22.6 ± 0.78°C	27.7 ± 0.49°C	33.9 ± 0.55°C
**Fig. 1D**	**Observation**	MCT1	WT	24.6 ± 0.32°C	28.1 ± 0.21°C	31.7 ± 0.50°C
		MCT2	WT	24.6 ± 0.30°C	28.0 ± 0.30°C	31.7 ± 0.52°C
**Fig. 1D**	**Gene expression**	MCT1	WT	24.6 ± 0.33°C	28.1 ± 0.20°C	31.7 ± 0.47°C
		MCT2	WT	24.7 ± 0.24°C	28.1 ± 0.22°C	31.6 ± 0.24°C
		MCT3	WT	25.0 ± 0.28°C	28.2 ± 0.17°C	31.2 ± 0.11°C
		MCT4	WT	24.0 ± 0.29°C	28.0 ± 0.17°C	31.7 ± 0.25°C
**Fig. 2B**	**Experiment 1**	MCT	12Rev	24.2 ± 0.33°C	28.1 ± 0.20°C	32.3 ± 0.07°C
		MCT	12Del	23.8± 0.33°C	27.8 ± 0.41°C	32.5 ± 0.32°C
**Fig. 2B**	**Experiment 2**	MCT	12Rev	24.8 ± 0.32°C	28.2 ± 0.19°C	32.1 ± 0.10°C
		MCT	12Del	24.2± 0.21°C	27.9 ± 0.20°C	32.5 ± 0.49°C
**Fig. 2B**	**Experiment 3**	MCT	12Rev	24.5 ± 0.11°C	27.4 ± 0.16°C	32.4 ± 0.24°C
		MCT	12Del	24.1± 0.16°C	27.7 ± 0.16°C	32.9 ± 0.15°C
**Fig. 2C**	**Observation**	MCT	12Rev	23.6 ± 0.16°C	27.6 ± 0.26°C	32.3 ± 0.24°C
		MCT	12Del	23.8 ± 0.35°C	28.2 ± 0.18°C	32.5 ± 0.20°C
**Fig. 2C**	**Viral load**	MCT1	12Rev	24.5 ± 0.20°C	27.9 ± 0.17°C	32.3 ± 0.38°C
		MCT2	12Rev	23.6 ± 0.35°C	28.5 ± 0.48°C	32.4 ± 0.20°C
		MCT1	12Del	24.8 ± 0.21°C	28.1 ± 0.17°C	32.2 ± 0.23°C
		MCT2	12Del	24.9 ± 0.21°C	28.1 ± 0.18°C	32.2 ± 0.22°C
**Fig. 4**	**Experiment 1**	MCT	WT/ CT	23.5 ± 0.22°C	28.2 ± 0.31°C	32.6 ± 0.10°C
		MCT	WT/ anti-TNF-α	23.8 ± 0.29°C	28.5 ± 0.24°C	33.0 ± 0.27°C
	**Experiment 2**	MCT	WT/ CT	24.4 ± 0.44°C	28.3 ± 0.43°C	32.8 ± 0.46°C
		MCT	WT/ anti-TNF-α	24.2 ± 0.48°C	28.3 ± 0.53°C	32.6 ± 0.72°C

Supplemental Experimental Procedures

Production and characterization of CyHV-3 ORF12 recombinant strains

CyHV-3 ORF12 recombinant strains were produced by using BAC cloning and prokaryotic recombination technologies (Figure S1). The parental plasmid was the CyHV-3 FL BAC clone (Boutier et al., 2015b). Recombinant plasmids were produced by using galactokinase (*galK*) positive/negative selection in bacteria (Boutier et al., 2015b) (Figure S1A) before reconstitution of infectious virus by transfection into CCB cells. The first recombination process (*galK* positive selection) replaced ORF12 by *galK*, resulting in the FL BAC ORF12 Del *galK* plasmid. Recombination was achieved by using the H1-*galK*-H2 recombination cassette, which consisted of the *galK* gene flanked by 50 bp sequences of the CyHV-3 genome flanking ORF12. This recombination cassette was produced by PCR by using the p*galK* vector as a template and the primers listed in the Table below. The second recombination process (*galK* negative selection) removed the *galK* gene (FL BAC ORF12 Del plasmid) or replaced the *galK* gene by the CyHV-3 wild type ORF12 sequence (FL BAC ORF12 Rev plasmid). The FL BAC ORF12 Del plasmid was obtained by recombination with the H1-H2 cassette. This cassette consisted of 50 bp of the CyHV-3 genome upstream and downstream of ORF12. The FL BAC ORF12 Rev plasmid was produced by recombination with the H1-ORF12-H2 cassette. This cassette was produced by PCR using the primers listed in the Table below and CyHV-3 FL DNA as the template. To reconstitute infectious virus, the recombinant BAC plasmids were co-transfected with the pGEMT-TK plasmid (molecular ratio of 1:75) in CCB cells. Transfection with the pGEMT-TK plasmid induced recombination upstream and downstream of the BAC cassette, leading to its complete removal and consequent reversion to a wild-type ORF55 locus (FL BAC revertant strains). Plaques negative for enhanced green fluorescent protein (EGFP) expression (encoded by the BAC cassette) were picked and amplified.

The structure (Figure S1C) and transcription (Figure S1D) of the ORF12 region of CyHV-3 recombinants were characterized by Southern blot and RT-PCR analyses, respectively. Probes for Southern blot analyses were produced by PCR using the primers listed in the Table below. Transcriptional analyses by RT-PCR were performed as described previously (Boutier et al., 2015b) with the primers listed in the Table below.

For genetic characterization of CyHV-3 recombinants by full length genome sequencing, DNA (500 ng) was sheared by sonication to an average size of 400 bp and prepared for sequencing by using a KAPA library preparation kit (KAPA Biosystems). The fragments were A-tailed, ligated to the NEBnext Illumina adaptor (New England Biolabs), and amplified by PCR. Index tags were added by six cycles of PCR using KAPA HiFi HotStart and NEBnext indexing primers. The libraries were analyzed by using a MiSeq DNA sequencer running v2 chemistry (Illumina). Approximately 1 million 250-nucleotide paired-end reads were obtained per sample. The reads were prepared for assembly by using Trim Galore v. 0.2.2 (http://www.bioinformatics.babraham.ac.uk/projects/trim_galore). Sequence accuracy was checked by using BWA v. 0.6.2-r126 (Li and Durbin, 2010) to assemble the reads against the sequence of the parental strain (KP343683) adapted by the conceptual mutagenesis performed and visualizing the alignment by using Tablet v. 1.13.08.05 (Milne et al., 2013). The 12Rev virus exhibited a sequence identical to the parental sequence. As intended, the 12Del virus completely lacks ORF12 (29795-30181). It also contains two nucleotide substitutions, the first a G to A transition at 240846 and the second an A to G transition at 58571. The former is a synonymous substitution in ORF139, and the second a nonsynonymous substitution in ORF36 resulting in a N to D change, a polymorphism found in CyHV-3 and in the orthologue of CyHV-2. In all viral genomes, two regions were of undetermined length: an A repeat and a GA repeat located at 32540-32465 and 177568-177730 nt, respectively, in the CyHV-3 reference sequence (NC_009127.1).

The fitness of the CyHV-3 recombinants to replicate in cell culture was investigated by multi-step growth curves as described previously (Boutier et al., 2015b). Briefly, cultures of CCB cells were inoculated in triplicate with CyHV-3 at a MOI of 0.1 pfu/cell. Supernatants were collected from the infected cultures at successive intervals and stored at -80°C. Titration of infectious viral particles was determined by duplicate plaque assays.

TNF-α binding assay

Binding of carp TNF-α to the CyHV-3 secretome was analyzed by ELISA (Forlenza et al., 2009). ELISA plates were coated with 5 µg total protein from concentrated CCB supernatants. After blocking, plates were incubated with the supernatants from EPC cells that had been transfected previously with pTNF-α1, pTNF-α2 or pEmpty, or with purified carp TNF-α1/TNF-α2 expressed in bacteria (Forlenza et al., 2009). Bound TNF-α was quantified by using two detection systems: rabbit anti-TNF-α antibodies followed by goat anti-rabbit IgG-HRP, or mouse monoclonal (MAb) anti-His antibody (Quiagen) followed by goat anti-mouse IgG-HRP (Forlenza et al., 2009).

TNF-α bioluminescent reporter assay

EPC cells stably transfected with pNiFty2-Luc (InvivoGen) (Piazzon et al., 2015), hereafter referred to as EPC-NFκB-Luc cells, were used to measure TNF-α bioactivity. Supernatants from EPC cells, which had been transfected previously with pTNF-α1, pTNF-α2 or pEmpty (Forlenza et al., 2009), were pre-incubated with concentrated supernatants of CyHV-3-infected or mock-infected CCB cells (40 µg total protein) for 30 min at RT to allow ORF12-TNF-α binding. EPC-NFκB-Luc cells seeded in 96-well plates were stimulated with 50 µl of the pre-incubated mixtures described above. After incubation for 6 h at 27°C, cells were lysed and bioluminescence was measured. The fold change of luminescence was calculated by dividing the light units obtained for each sample by the result obtained for the respective control sample.

Tank systems

A single chamber tank (SCT) system and a multi-chamber tank (MCT) system were used in this study (Figure 1A; see also Supplemental Information M1). The SCT system consisted of an 80 L single compartment tank (width x depth x height: 0.5 x 0.4 x 0.4 m) with a mean constant temperature of approximately 24, 28 or 32°C. The MCT system consisted of a 144 L tank (width x depth x height: 1.2 x 0.3 x 0.4 m) subdivided into three equal compartments by two rigid thermal insulation panels (6 cm thick and made of polyurethane foam). Neighboring compartments were connected by a 0.4 m long transparent tunnel with a square cross-section (8 x 8 cm) placed at the bottom of the tank. Each of the three chambers had independent aeration, circulation and filtering systems. A thermal gradient (24°C - 28°C - 32°C) was established between the three chambers by cooling the first compartment and by increasing heating of the second and third compartments. Temperatures in all three compartments were controlled by measurements every 30 min. The observed temperatures of the MCTs used for the experiments in this manuscript are presented as the mean ± SD in Table S1. To simplify reading of the manuscript, the theoretical gradient of 24°C - 28°C - 32°C is presented in the manuscript using the color code described in the legend of Figure 1. The MCTs were placed in the experimental room as pairs around a central axis of symmetry, with the 32°C compartments being close to this axis and the 24°C compartments being furthest away. The positions of the experimental groups were swapped systematically between replicate tanks. In the MCTs, daily feeding was performed in the 24°C compartment independently of the position of the fish.

Monitoring of fish position in the MCTs

Fish (n=15) were initially introduced into the 24°C compartment of the MCT, in which the tunnels were obstructed by grids to prevent migration out of the compartment. After an acclimatization period of 3 weeks, the grids were removed, and the distribution of fish into the three compartments was monitored over time. A digital camera (Logitech HD Webcam c310) placed in front of each MCT recorded pictures every 3 min. The number of fish in each compartment was counted manually from the images captured at each successive 30 min, resulting in 48 measurements per day. When the positions of the fish were not compatible with an accurate count, measurements were made from the previous or following image (i.e. collected 3 min before or after image examined initially). The results are presented as the mean + SD (n=48) of the number of fish observed per day in each compartment.

Statistical analyses

Most statistical analyses were performed by using linear models. The observations on fish position in MCTs (Figure 1B and D, Figure 2B and Figure 4) were analyzed by using daily averages of the number of fish present in each compartment for each replicate as follows: a mixed model was set up with these averages as dependent variables and the status (WT or Mock, Figure 1B and D; 12Rev or 12Del, Figure 2B; and Ctrl or anti-TNF-α, Figure 4), the time and the interaction between time and status as independent variables. Due to the lack of some observations (e.g. on the day of infection or at the end of the observation period or upon deaths), averages might have been computed over unequal number of observations, requiring a weighting of the data (using the corresponding number of observations) in order to estimate the standard errors correctly. Potential correlations between successive measurements over the same set of fish were taken into account by a type 1 auto-regressive structure using an Akaike Information Criterion comparison. Obtained estimators were then globally compared using type 3 tests of the fixed effects of the model with a Kenward-Roger correction for the degrees of freedom. Post-hoc comparisons of the sets of fish (WT or Mock, Figure 1B and D; 12Rev or 12Del, Figure 2B; and Ctrl or anti-TNF-α, Figure 4) were then obtained for each day of the experiment, testing the null hypothesis that the means are actually equal. Differences in viral load (data after log transformation) and cytokine expression (data after log transformation) (Figure 1D) between CyHV-3 infected fish collected at various post-infection times and mock-infected fish sampled at day 0 were analyzed by using a one-way ANOVA. Post-hoc comparisons between days were performed by using Tukey's test. These analyses were performed independently for each cytokine. The TNF-α binding assay (Figure 3C

and Figure S2), TNF-α neutralization assay (Figure 3D), virus load measurement (Figure 2A and C), and multi-step growth curves (Figure S1E) were analyzed by using two- or three-way ANOVA. More precisely, for the binding assay of TNF-α, the level of binding was modeled by using a linear model involving cell supernatants, TNF-α1 or TNF-α2 volumes as well as their interactions. For the neutralization assay of TNF-α biological activity, the fold change of luminescence (ratio of the TNF-α1 or TNF-α2 levels respective to the control supernatant level) was modeled by using a linear model involving cell supernatants, cytokine (TNF-α1, TNF-α2 or control) supernatants, and their interactions. For viral load, the viral genome copies (data after log transformation) were modeled by using a linear model involving virus strains, tanks, and their interactions. For multi-step growth curves, the logarithm of the titre was modeled by using a linear model involving the day, viral strains, temperature, and their interactions. For the survival analyses (Figure 1C and 2A), a standard SAS LIFETEST procedure was performed. Survival curves were compared pairwise. Statistical significance is represented as follows: -, not significant; *, $p<0.05$; **, $p<0.01$; ***, $p<0.001$; and ****, $p<0.0001$. P-values <0.05 were considered significant.

Primer sets used in this study (related to Supplemental Experimental Procedures)

	Primer name	Sequence (5'- 3')	Coordinates*/ accession number
**Synthesis of recombination cassettes**			
**Cassette name**			
H1-*galK*-H2	ORF12 *galK* F	AGGCTGCACTGCTGCGCACAGTGACGAGTAGACGGTGGGA GGTCGGTGAACCTGTTGACAATTAATCATCGGCA	29745-29794
	ORF12 *galK* R	CTTGTTTTACTATACTCTATCACATCTCCGACTTGATTTCTT CTCAAACCTCAGCACTGTCCTGCTCCTT	30231-30182
H1-ORF12-H2	ORF12 Fw RVT	ATGAAGAGTTTGTGTCGAGC	29668-29687
	ORF12 Rev RVT	GGCTACGTATAACTGTCATG	30312-30293
**Synthesis of probes for Southern blot analysis**			
**Probe name**			
CyHV-3 ORF55	ORF55InF	AGCGCTACACCGAAGAGTCC	95990-96009
	ORF55stopR	TCACAGGATAGATATGTTACAAG	96516-96494
CyHV-3 ORF12 Del	ORF12 Int Fw 5'	TCGTAGTCGCCCTGACATCC	29847-29866
	ORF12 Int Rev 3'	AACTAGACACTCATCATGCGG	30122-30102
CyHV-3 ORF12 external	ORF12 Fw 5'	GAATTTATATGCAGCGAGTG	29723-29742
	ORF12 Rev 3'	TATTGTCTGTTTCTGTGCTC	30261-30242
**Transcriptional analysis**			
**Gene amplified**			
CyHV-3 ORF11	ORF11 Fw	CAACCCACAACAGCAGTACC	29595-29576
	ORF11 Rev	TTGCCCTGTCTCATCTTGGT	29374-29393
CyHV-3 ORF12	ORF12 Int Fw 5'	TCGTAGTCGCCCTGACATCC	29847-29866
	ORF12 Int Rev 3'	AACTAGACACTCATCATGCGG	30122-30102
CyHV-3 ORF13	ORF13 Fw	TGTGAGTCATGACAGTTATACGT	30286-30308
	ORF13 Rev	ATGACTGACTGGACATCGGC	30503-30484
CyHV-3 ORF55	ORF55 ATG Fw	ATGGCTATGCTGGAACTGG	95866-95884
	ORF55 In Rev	GGCGCACCCAGTAGATTATG	96467-96448
Carp *β-actin*	Actin-Fw	ATGTACGTTGCCATCCAGGC	M24113
	Actin-Rev	GCACCTGAACCTCTCATTGC	
**qPCR analysis for quantification of viral load**			
**Gene amplified**			
CyHV-3 ORF89	KHV-86F	GACGCCGGAGACCTTGTG	AF411803
	KHV-163R	CGGGTTCTTATTTTTGTCCTTGTT	
	KHV-109P	(6FAM) CTTCCTCTGCTCGGCGAGCACG (BHQ1)	
Carp *glucokinase*	CgGluc-162F	ACTGCGAGTGGAGACACATGAT	AF053332
	CgGluc-230R	TCAGGTGTGGAGCGGACAT	
	CgGluc-185P	(6FAM) AAGCCAGTGTCAAAATGCTGCCCACT (BHQ1)	
**RT-qPCR analysis for quantification of cytokine expression**			
**Gene amplified**			
Carp *40S*	40S-F	CCGTGGGTGACATCGTTACA	AB012087
	40S-R	TCAGGACATTGAACCTCACTGTCT	
Carp *il1β*	IL-1β-F	AAGGAGGCCAGTGGCTCTGT	AJ245635
	IL-1β-R	CCTGAAGAAGAGGAGGCTGTCA	
Carp *TNF-α1* and *TNF-α2*	TNF-α1 and 2-F	GCTGTCTGCTTCACGCTCAA	AJ311800 and AJ311801
	TNF-α1 and 2-R	CCTTGGAAGTGACATTTGCTTTT	
Carp *il6a*	IL-6a-F	CAGATAGCGGACGGAGGGGC	KC858890
	IL-6a-R	GCGGGTCTCTTCGTGTCTT	
Carp *il6b*	IL-6b-F	GGCGTATGAAGGAGCGAAGA	KC858889
	IL-6b-R	ATCTGACCGATAGAGGAGCG	

*Coordinates based on the reference CyHV-3 genome (Accession number: NC_009127.1)
Underlined: 50 bp corresponding to the CyHV-3 sequence. Red: sequence corresponding to *galK*

Supplemental References

Li, H., and Durbin, R. (2010). Fast and accurate long-read alignment with Burrows-Wheeler transform. Bioinformatics *26*, 589-595.

Milne, I., Stephen, G., Bayer, M., Cock, P.J., Pritchard, L., Cardle, L., Shaw, P.D., and Marshall, D. (2013). Using Tablet for visual exploration of second-generation sequencing data. Brief. Bioinform. *14*, 193-202.
