# Supplementary figures and images for: Conserved Fever Pathways across Vertebrates: A Herpesvirus Expressed Decoy TNF-α Receptor Delays Behavioral Fever in Fish
Source: Cell Host Microbe. 2017 Feb 8;21(2):244–53. doi: 10.1016/j.chom.2017.01.010 (PMC5301049; doi:10.1016/j.chom.2017.01.010)

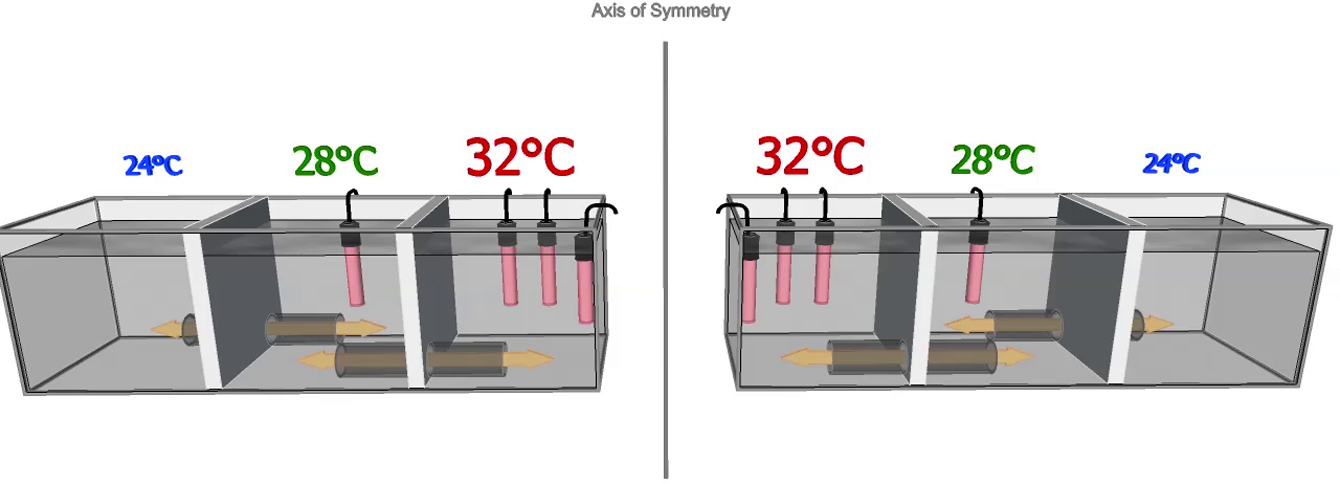

Supplement: Movie S1. Common Carp Express Behavioral Fever in Response to CyHV-3 Infection, Related to Figure 1B — This movie starts with an animated cartoon illustrating the structure of the MCT used in this study and how the MCTs were positioned in the room along a central axis of symmetry (warmest compartments being the closest to the center of the room). The video then presents movies of fish that were mock-infected (left and first presented MCT) or infected (right and second presented MCT) 7 days earlier with wild-type CyHV-3. [file mmc2.jpg]

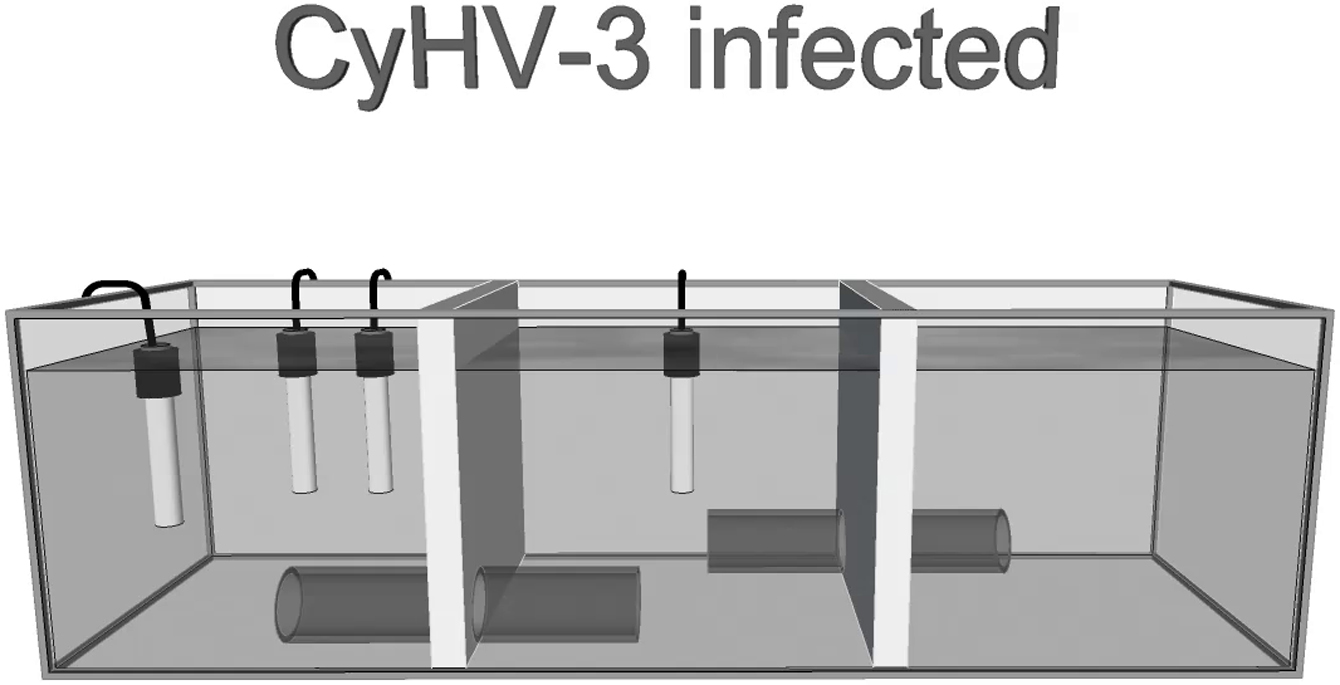

Supplement: Movie S2. Effect of Water Temperature on the Development of CyHV-3 Disease, Related to Figure 1C — This video starts with an animated cartoon illustrating the structure of a MCT in which the tunnels were blocked by grids. The video then presents a movie of fish that were infected 9 days earlier with wild-type CyHV-3 before their distribution into the compartments of the MCT. [file mmc3.jpg]

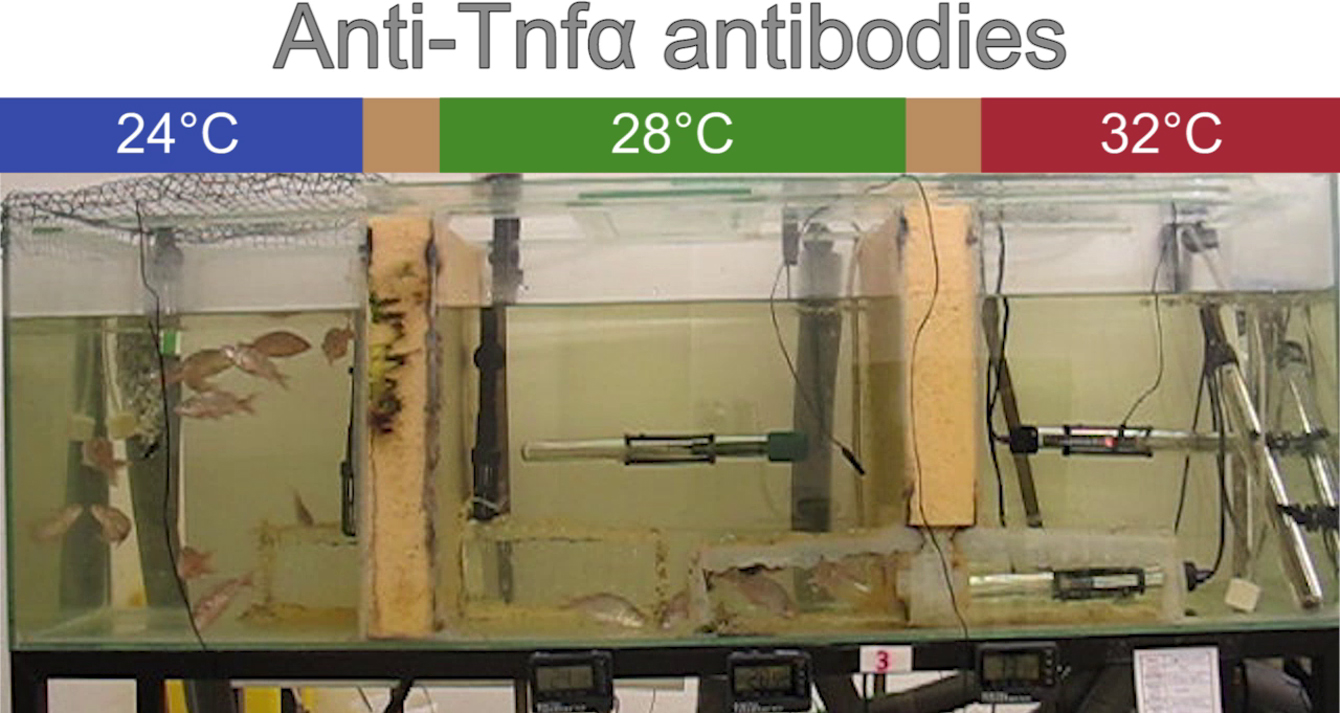

Supplement: Movie S3. Anti-TNF-α Antibodies Inhibit the Expression of Behavioral Fever Induced by CyHV-3 Infection, Related to Figure 4 — This video starts with an animated cartoon explaining the flow chart of this experiment. Fish were first infected with CyHV-3 and then injected 3 days later with anti-TNF-α antibodies (left and first presented MCT) or irrelevant control antibodies (right and second presented MCT). Movies of the fish were recorded at 6 dpi. [file mmc4.jpg]
