# Supplementary material for: Conserved Fever Pathways across Vertebrates: A Herpesvirus Expressed Decoy TNF-α Receptor Delays Behavioral Fever in Fish
Source: Cell Host Microbe. 2017 Feb 8;21(2):244–53. doi: 10.1016/j.chom.2017.01.010 (PMC5301049; doi:10.1016/j.chom.2017.01.010)
Supplement: Document S2. Article plus Supplemental Information [file mmc5.pdf]

Short Article

Cell Host & Microbe

Conserved Fever Pathways across Vertebrates: A Herpesvirus Expressed Decoy TNF-α Receptor Delays Behavioral Fever in Fish

Graphical Abstract

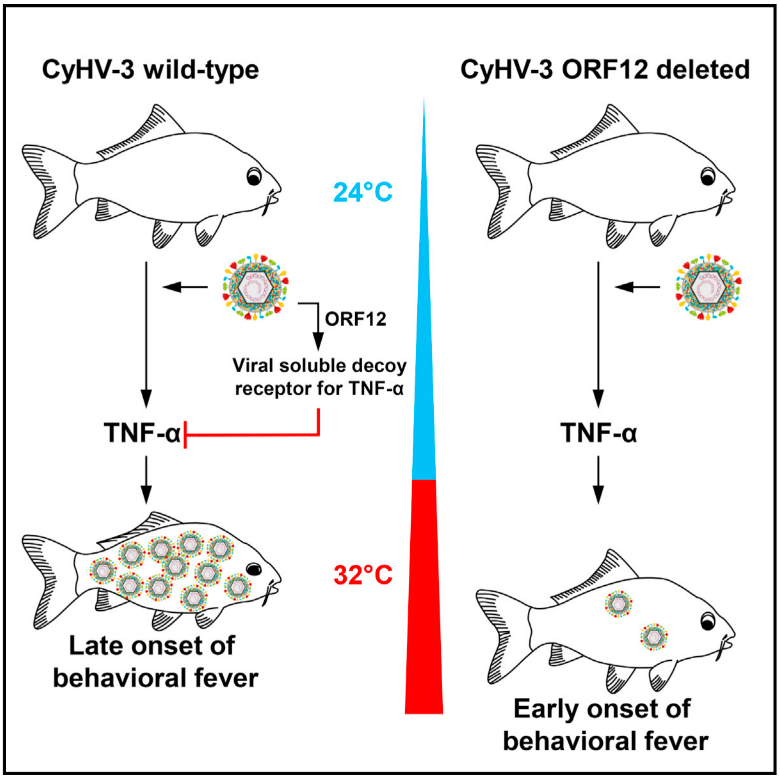

Highlights

- Behavioral fever exhibited by carp in response to CyHV-3 infection is host beneficial
- CyHV-3 ORF12 delays behavioral fever expression, thereby promoting its own replication
- CyHV-3 ORF12 encodes a soluble decoy receptor for TNF-α
- TNF-α is a mediator of behavioral fever expressed by CyHV-3 infected carp

Authors

Krzysztof Rakus, Maygane Ronsmans, Maria Forlenza, ..., Thomas Michiels, Geert F. Wiegertjes, Alain Vanderplasschen

Correspondence

a.vdplasschen@ulg.ac.be

In Brief

Ectotherms can express behavioral fever to limit pathogen infection. Rakus et al. show that a carp herpesvirus delays behavioral fever by expressing a soluble decoy receptor for TNF-α, thus promoting its own replication. In addition, they demonstrate a role for TNF-α in the induction of behavioral fever in teleost fish.

Rakus et al., 2017, Cell Host & Microbe *21*, 244–253
February 8, 2017 © 2017 The Author(s). Published by Elsevier Inc.
http://dx.doi.org/10.1016/j.chom.2017.01.010

Cell Host & Microbe
Short Article

Conserved Fever Pathways across Vertebrates: A Herpesvirus Expressed Decoy TNF-α Receptor Delays Behavioral Fever in Fish

Krzysztof Rakus,[1,2,11] Maygane Ronsmans,[1,11] Maria Forlenza,[3] Maxime Boutier,[1] M. Carla Piazzon,[3] Joanna Jazowiecka-Rakus,[1,4] Derek Gatherer,[5] Alekos Athanasiadis,[6] Frédéric Farnir,[7] Andrew J. Davison,[8] Pierre Boudinot,[9] Thomas Michiels,[10] Geert F. Wiegertjes,[3] and Alain Vanderplasschen[1,12,*]
[1]Immunology-Vaccinology, FARAH, Faculty of Veterinary Medicine, University of Liège, Liège 4000, Belgium
[2]Department of Evolutionary Immunology, Institute of Zoology, Jagiellonian University, Krakow 30-387, Poland
[3]Cell Biology and Immunology Group, Department of Animal Sciences, Wageningen University and Research, Wageningen 6708WD, the Netherlands
[4]Maria Sklodowska-Curie Memorial Cancer Center and Institute of Oncology, Gliwice 44-101, Poland
[5]Division of Biomedical and Life Sciences, Faculty of Health and Medicine, Lancaster University, Lancaster LA1 4YW, UK
[6]Instituto Gulbenkian de Ciência, Oeiras 2781-156, Portugal
[7]Biostatistics and Bioinformatics, FARAH, University of Liège, Liège 4000, Belgium
[8]MRC - University of Glasgow Centre for Virus Research, Glasgow G61 1QH, UK
[9]Virologie et Immunologie Moléculaires, INRA, Jouy-en-Josas 78352, France
[10]de Duve Institute, Université Catholique de Louvain, Brussels 1200, Belgium
[11]Co-first author
[12]Lead Contact
*Correspondence: a.vdplasschen@ulg.ac.be
http://dx.doi.org/10.1016/j.chom.2017.01.010

SUMMARY

Both endotherms and ectotherms (e.g., fish) increase their body temperature to limit pathogen infection. Ectotherms do so by moving to warmer places, hence the term "behavioral fever." We studied the manifestation of behavioral fever in the common carp infected by cyprinid herpesvirus 3, a native carp pathogen. Carp maintained at 24°C died from the infection, whereas those housed in multi-chamber tanks encompassing a 24°C–32°C gradient migrated transiently to the warmest compartment and survived as a consequence. Behavioral fever manifested only at advanced stages of infection. Consistent with this, expression of CyHV-3 ORF12, encoding a soluble decoy receptor for TNF-α, delayed the manifestation of behavioral fever and promoted CyHV-3 replication in the context of a temperature gradient. Injection of anti-TNF-α neutralizing antibodies suppressed behavioral fever, and decreased fish survival in response to infection. This study provides a unique example of how viruses have evolved to alter host behavior to increase fitness.

INTRODUCTION

When infected by pathogens, endotherms and ectotherms can both increase their body temperature to limit the infection. In endotherms, this cardinal response to infection is called fever (for a recent review, see Evans et al., 2015). It relies mainly on thermogenesis, and also on physiological and behavioral modifications leading to reduced heat loss by the body. With the exception of a few rare examples, ectotherms lack intrinsic thermogenesis and so have a body temperature very close to the temperature of the environment. In a temperature gradient, ectotherms select a species-specific range of preferred temperature, which is defined as final thermal preferendum (FTP) (for a recent review, see Rakus et al., 2017). In response to infection or injection of exogenous pyrogens, ectotherms can increase their body temperature above their FTP through migration to warmer environments. This phenomenon is known as behavioral fever and is defined as an acute increase of the FTP consecutive to an infection (Rakus et al., 2017). Behavioral fever has been reported in a broad range of ectotherms including vertebrates (fishes, amphibians, and reptiles) and invertebrates.

Regulation of behavioral fever in ectotherms is evolutionarily related to fever in endotherms at various levels of the relevant regulatory pathways (Evans et al., 2015; Rakus et al., 2017), including the roles of exogenous pyrogens as inducers, the importance of the hypothalamic preoptic area as an integration site for pyrogenic signals, and the key role of prostaglandins as effector mediators. However, no study has yet determined whether this evolutionary relationship extends to the endogenous pyrogens, namely the cytokine mediators that inform the brain of exogenous pyrogens detected by immune cells throughout the body. In endotherms, cytokines such as interleukin 1β (IL-1β), IL6, tumor necrosis factor α (TNF-α), and interferons (Dinarello, 1999; Netea et al., 2000) have been shown to act as endogenous pyrogens.

At least in some infectious models, fever in endotherms and behavioral fever in ectotherms can increase host survival (Evans et al., 2015; Rakus et al., 2017). This beneficial effect is the

 © 2017 The Author(s). Published by Elsevier Inc.
This is an open access article under the CC BY license (http://creativecommons.org/licenses/by/4.0/).

consequence of the elevation of body temperature, which enhances the efficiency of both innate and (when existing) adaptive immune mechanisms and can restrict replication of invading pathogens. Through the expression of dedicated genes, pathogens are able to manipulate virtually all the physiological processes of their host that can affect their replication and transmission. However, to date, there is no report of a pathogen being able to affect the expression of behavioral fever by its host.

Cyprinid herpesvirus 3 (CyHV-3) is the causative agent of a lethal, highly contagious and notifiable disease in common and koi carp (*Cyprinus carpio*) (Boutier et al., 2015a). The outcome of CyHV-3 infection is highly dependent on temperature both in vitro and in vivo, with temperatures between 18°C and 28°C allowing viral replication in vitro and development of CyHV-3 disease in vivo, whereas temperatures above 30°C rapidly block CyHV-3 replication and the development of CyHV-3 disease (Gilad et al., 2003). During our studies of CyHV-3 pathogenesis (Boutier et al., 2015b), we observed that carp infected at 24°C (within the FPT of healthy carp) tended to concentrate around the tank heater when it was running. This observation led us to hypothesize that infected subjects might express behavioral fever in natural environments where temperature gradients exist (Boehrer and Schultze, 2008).

Here, we used the infection of carp by CyHV-3 as a homologous virus-host model to study the expression of behavioral fever. We demonstrate the ability of this virus to alter this behavior of its host through the expression of a single gene and identify the role of TNF-α as a mediator of behavioral fever in ectotherms.

RESULTS

Carp Express Behavioral Fever in Response to CyHV-3 Infection

First, we tested the hypothesis that common carp express behavioral fever in response to CyHV-3 infection. Carp were housed in multi-chamber tanks (MCTs; Figure 1A) where they could freely move between three compartments maintained at 24°C, 28°C, and 32°C. Fish distribution in the three compartments was recorded over time (Figure 1B). In the absence of infection (days −5 to 0), in all six observed MCTs, the majority of carp were distributed in the 24°C compartment and to a lesser extent in the 28°C compartment. At day 0, the fish in three MCTs were infected with CyHV-3, while the three remaining MCTs were mock-infected. Between 4 and 6 days post-infection (dpi), infected fish began to reside more frequently in the 32°C compartment. This observation is also illustrated in the supplemental file Movie S1, consisting of a short video shot at 7 dpi (MCTs of experiment 1). The number of infected fish in the 32°C compartment peaked at around 7–9 dpi. By 13 dpi, the distribution of fish returned to normal, with only the occasional fish in the 32°C compartment (Figure 1B). Global statistical analyses of the data from the three replicates demonstrated that carp infected by CyHV-3 express behavioral fever as revealed by a significantly higher number of fish in the 32°C compartment ($p < 0.001$) between 6 to 11 dpi. Interestingly, none of the fish infected with CyHV-3 in the MCTs died during the course of these experiments, suggesting that expression of behavioral fever could be beneficial for CyHV-3 infected carp.

Expression of Behavioral Fever Is Beneficial for CyHV-3 Infected Carp

Carp were distributed in single chamber tanks (SCT; Figure 1A) maintained at 24°C, 28°C, or 32°C, or in the different compartments of a MCT in which the tunnels between the compartments were blocked by grids (MCT-blocked) or in a MCT. At time 0, all fish were infected with CyHV-3 (Figure 1C). Survival rates of 0% and 20% were recorded in SCTs maintained at 24°C and 28°C, respectively. Significantly higher survival rates of 40% ($p = 0.033$) and 80% ($p = 0.004$) were observed when infected fish were blocked by grids in the 24°C and 28°C compartments of the MCT, respectively. Consistent with an earlier report (Gilad et al., 2003), infected fish in the SCT at 32°C or blocked in the 32°C compartment of the MCT did not develop CyHV-3 disease. The effect of temperature on the development of CyHV-3 disease is also illustrated by Movie S2. It shows that clinical signs expressed by fish blocked in MCT compartments were inversely related to temperature. Importantly, all the fish infected in the MCTs survived the infection as the consequence of the expression of behavioral fever.

Expression of Behavioral Fever Occurs at an Advanced Stage of CyHV-3 Disease

The data above demonstrate that expression of behavioral fever is beneficial for CyHV-3 infected carp. However, clinical observation of fish in the MCTs revealed that their migration to the warmest compartment occurred only at an advanced stage of the disease. To verify the relatively late onset of behavioral fever with respect to viral replication and cytokine upregulation, fish infected in MCTs were analyzed over time for viral load and carp proinflammatory cytokine gene expression. Data presented in Figure 1D confirmed that the onset of behavioral fever observed in this experiment at 6 dpi occurred days after systemic replication of the virus (significant at 3 dpi) and upregulation of proinflammatory cytokines (significant for *il1*β and *TNF-*α*1*/α*2* at 3 dpi and for all cytokines tested at 5 dpi).

The relatively late onset of behavioral fever led us to postulate that this phenomenon might be delayed by the virus in order to retain its host at a temperature compatible with viral replication. As some viruses have been shown to express soluble decoy cytokine receptors (Epperson et al., 2012), and as the CyHV-3 genome potentially encodes such receptors (Yi et al., 2015), we hypothesized that CyHV-3 might express decoy receptor(s) able to neutralize putative pyrogenic cytokines produced by fish. CyHV-3 ORF12 was selected as a candidate because it encodes a putative soluble TNF-receptor homolog that is the most abundant viral protein of the CyHV-3 secretome (Ouyang et al., 2013).

CyHV-3 ORF12 Deletion Does Not Affect Viral Replication In Vitro or Virulence In Vivo under Standard Laboratory Conditions—SCT at 24°C

To investigate whether CyHV-3 is able to delay the expression of behavioral fever through expression of ORF12, a CyHV-3 ORF12 deletion (12Del) mutant and a revertant (12Rev) virus (in which ORF12 was restored) were derived from the parental wild-type (WT) strain (Figure S1). The structure and transcription of the ORF12 region, as well as the full-length genome sequences, were validated in all viral strains (Supplemental Information and

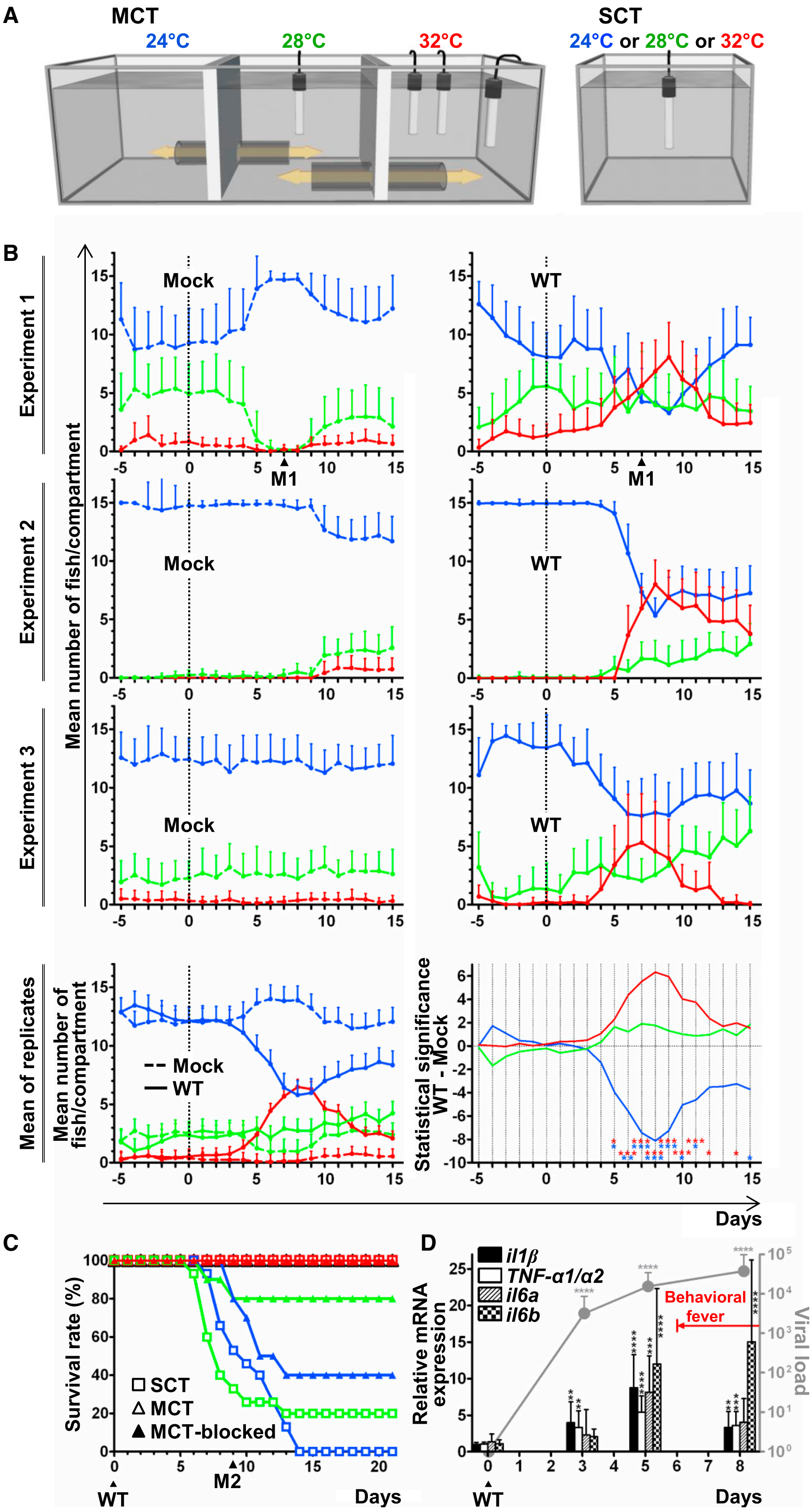

Figure 1. Carp Express Beneficial Behavioral Fever in Response to CyHV-3 Infection

(A) Experimental setup tank systems used: multi-chamber tank (MCT) and single-chamber tank (SCT). Unless stated in the figure, in vivo experiments were performed in MCTs. In this and subsequent figures, a color code was adopted to illustrate temperature, with blue, green, and red representing 24°C, 28°C, and 32°C, respectively. See also Table S1.

(B) Carp (n = 15 per tank) were housed in 6 MCTs. On day 0, fish were infected with wild-type CyHV-3 (WT) (right column, 3 upper graphs) or were mock-infected (Mock) (left column, 3 upper graphs). The number of fish in each compartment was counted every 30 min and expressed as a mean per day + SD. See also Movie S1 illustrating the first pair of MCTs at 7 dpi. The two lower graphs represent a global analysis of the data from the three replicates. The left graph presents the mean number + SD of fish in the different compartments of the MCTs based on the data of the three replicates. The statistical significance of the differences between the mean number of fish observed for WT infected and mock-infected groups is presented in the right graph. The days on which the number of fish per compartment was statistically different between WT infected and mock-infected fish are indicated according to the level of significance.

(C) The effect of temperature on survival rate after CyHV-3 infection. Carp were housed in SCTs (n = 15), in a MCT (n = 15) (MCT) and in each compartment (n = 10) of a MCT in which the tunnels were blocked by grids (MCT-blocked). Survival rates were measured according to time post-infection with CyHV-3 (WT) (see also Movie S2 recorded 9 dpi for the MCT-blocked).

(D) Carp (n = 15 per tank) were housed in 6 MCTs: 2 MCTs (1 mock-infected and 1 infected) were used for the observation of fish position, 4 MCTs were used for fish sampling (2 fish per time point per tank). At day 0, fish were mock-infected (1 MCT) or infected (5 MCTs) with CyHV-3 WT. At the indicated post-infection times, viral load (gray line) and cytokine gene expression were analyzed (n = 8; 2 fish were collected from each of the 4 replicate tanks leading to a total of 8 fish; mean + SD). Significant differences observed between CyHV-3 infected fish collected at different post-infection times and mock-infected fish are indicated by asterisks. The red arrow indicates the period during which expression of behavioral fever was significant ($p < 0.05$ or lower, significant increase of fish in the 32°C compartment).

Figure S1). Next, we characterized the phenotype of ORF12 deletion in vitro and in vivo. In cell culture, the three strains replicated comparably at 24°C and 28°C (Figure S1E). As reported earlier, CyHV-3 did not replicate at 32°C (Gilad et al., 2003). Since the WT and the 12Rev viruses exhibited identical genome sequences, only the latter was used in in vivo experiments. Inoculation of fish in SCTs at 24°C did not reveal a phenotype for 12Del different from that of the 12Rev (Figure 2A). Thus, similar clinical signs (data not shown) and survival rates (Figure 2A; left graph) were observed for 12Del and 12Rev. Moreover, viral load in gills increased similarly for the two viruses (Figure 2A; right graph). These observations demonstrate that ORF12 deletion does not exhibit a phenotype different from that of the 12Rev under standard laboratory conditions.

CyHV-3 ORF12 Delays the Expression of Behavioral Fever and Promotes CyHV-3 Replication in an Environment Encompassing a Temperature Gradient

In parallel to the experiments described above (Figure 2A), infections were also performed in MCTs (Figure 2B). Carp were distributed in 6 MCTs. At time 0, three MCTs were infected with 12Rev and the three others with 12Del. These triplicate experiments revealed that fish infected with 12Del expressed behavioral fever significantly earlier than those infected with 12Rev. Independently of the viral genotype, no fish died from CyHV-3 infection in MCTs, but because of their earlier migration to the warmest compartment, fish infected with 12Del expressed less severe clinical signs compared to fish infected with 12Rev. To verify this phenotypic difference between 12Del and 12Rev, which was not observed in SCTs at 24°C (Figure 2A), an independent infection with the two viruses was performed in 6 MCTs (3 MCTs per genotype), two intended for observation of fish distribution and four for sample collection, in order to measure viral load at various times post-infection (Figure 2C). In this experiment, fish infected with 12Del migrated 2 days earlier to the warmest compartment (Figure 2C; left graph). Interestingly, although the two viruses exhibited comparable replication kinetics in the SCTs (Figure 2A; right graph) and in the MCTs before expression of behavioral fever, they differed drastically once the fish migrated to the 32°C compartment (Figure 2C; right graph). Due to their earlier migration to a non-permissive temperature, fish infected with 12Del showed a faster and more drastic decrease of viral load than those infected with 12Rev. These results demonstrate that CyHV-3 is able to alter the expression of behavioral fever by its host via the expression of a single gene, thus favoring its replication.

CyHV-3 ORF12 Encodes a Soluble Decoy Receptor for TNF-α

Next, we tried to unravel the mechanism by which ORF12 delays expression of behavioral fever. According to our initial hypothesis, ORF12 might act as a soluble decoy receptor that neutralizes pyrogenic cytokines. As ORF12 encodes a putative soluble Tnf-receptor homolog, and as TNF-α is an endogenous pyrogen in endotherms (Dinarello et al., 1986), we investigated whether ORF12 encodes a soluble decoy receptor for TNF-α. The amino acid sequence of the ORF12 protein was aligned manually onto the human TNFR2 protein by using the Pfam TNFR_c6 domains (Finn et al., 2014) as a guide. TNFR2 has three TNFR_c6 domains in tandem and ORF12 has two, plus a fragment of a third. The solved structure of the human TNF-TNFR2 complex (3ALQ; Figure 3Ai) (Mukai et al., 2010) was used to develop a homology model of CyHV-3 ORF12 (Figure 3Aii). Superposition was achieved at 1.771Å (Figure 3Aiii) and favorably assessed by Ramachandran plot (Figure 3Aiv). The quality of the homology model strongly suggested that ORF12 forms a structure that contains at least two functional TNFR_c6 domains and that could bind TNF-α. The top BLASTP hit between ORF12 and eukaryotic proteins is the TNFR-2A from *Onchorhyncus mykiss* at 48% identity. The top hits with herpesvirus sequences are also in this range: UL144 in chimpanzee cytomegalovirus at 42% and ORF150D in cyprinid herpesvirus 1 at 40%.

To test the hypothesis that ORF12 encodes a functional soluble TNF-α receptor, concentrated supernatants were produced from cell cultures infected with CyHV-3 WT, 12Del, and 12Rev, as well as from mock-infected cultures. Silver staining of total supernatant proteins confirmed that ORF12 (predicted molecular mass 12.6 kDa) is the most abundant viral protein in the CyHV-3 secretome (Figure 3B). To test the ability of ORF12 to bind carp TNF-α, proteins of concentrated supernatants were coated on ELISA plates before incubation with carp TNF-α1 or TNF-α2 (Figure 3C). Carp TNF-α1 and TNF-α2 are both homologs of mammalian TNFSF2 (TNF-α) (Forlenza et al., 2009). Quantification of TNF-α binding demonstrated that the WT and 12Rev supernatants contained TNF-α-binding activity, in contrast to the 12Del and mock-infected supernatants (Figure 3C). These results were confirmed by independent ELISA combining a different detection system (anti-His-tag monoclonal antibody) and an alternative prokaryotic source of TNF-α1/ TNF-α2 (Figure S2). Next, a bioluminescence reporter assay was used to determine whether ORF12 binding to TNF-α could neutralize its ability to activate NF-κB signaling (Figure 3D). When incubated with concentrated supernatants from mock- or 12Del-infected cells, carp TNF-α1 and TNF-α2 induced activation of NF-κB similarly. This activation was completely inhibited when the cytokines were pre-incubated with WT or 12Rev supernatants (Figure 3D). These data demonstrated that ORF12 encodes a soluble TNF-α receptor able to neutralize both TNF-α1 and TNF-α2 from carp.

TNF-α Is a Mediator of Behavioral Fever Expressed by CyHV-3 Infected Carp

The ability of CyHV-3 ORF12 to delay the expression of behavioral fever, together with the results above showing that ORF12 encodes a decoy receptor able to neutralize TNF-α, led us to hypothesize that TNF-α might act as a pyrogenic cytokine in ectotherms, as it does in endotherms. To explore this hypothesis, carp were distributed in 4 MCTs and infected at time 0 with the CyHV-3 WT strain and then, 3 days later, injected intraperitoneally with control irrelevant or anti-TNF-α neutralizing antibodies (2 MCTs per condition; Figure 4). Injection of anti-TNF-α neutralizing antibodies induced a significant reduction of migration to the 32°C compartment, and as a consequence only 80% and 60% of the fish survived. By contrast, infected fish injected with irrelevant antibodies migrated efficiently to the 32°C compartment, and all survived the infection. This observation is also illustrated by Movie S3 recorded 6 dpi for the first replicate. Injection of anti-TNF-α neutralizing antibodies did not reduce

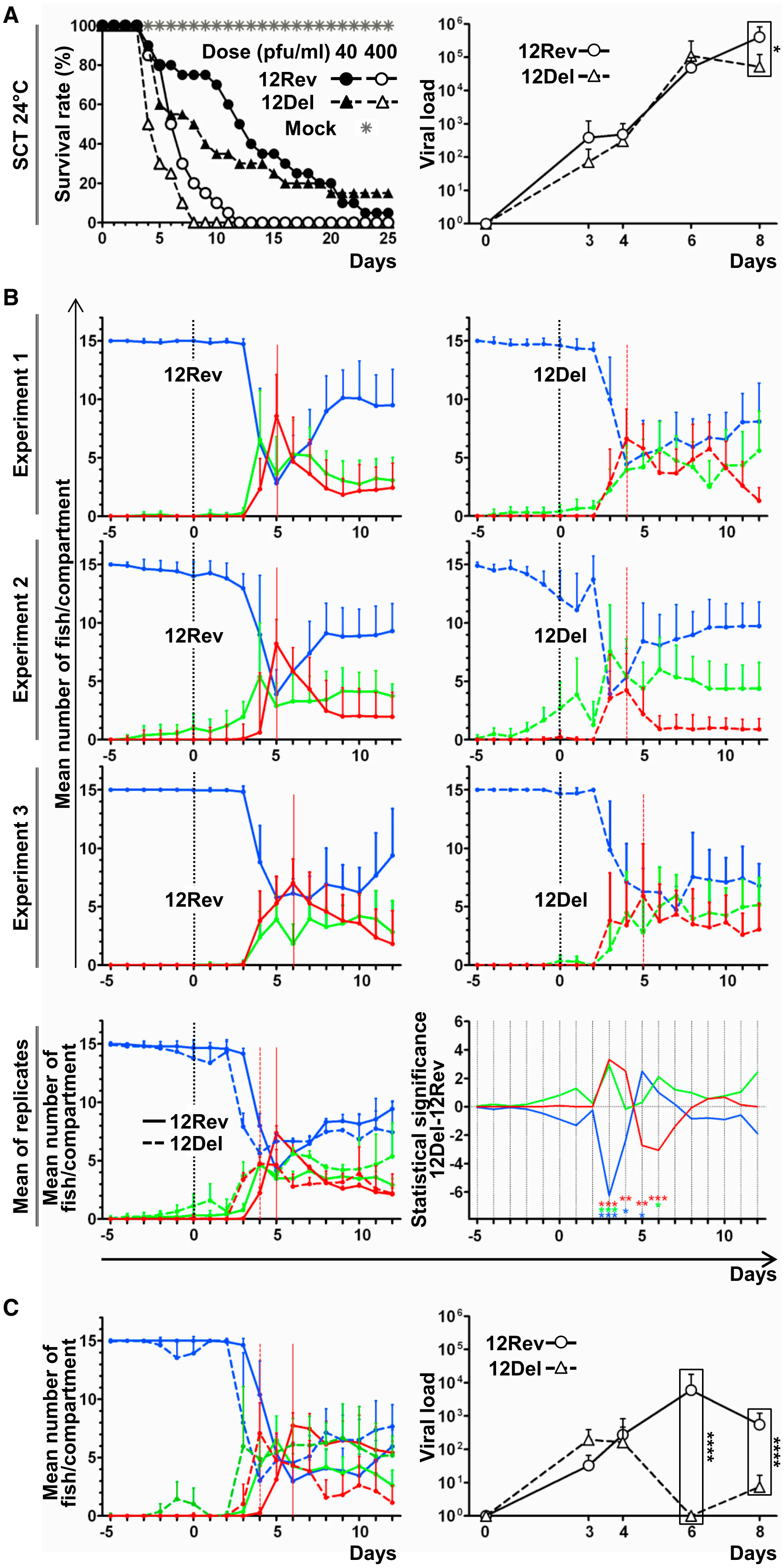

Figure 2. In Vivo Phenotyping of CyHV-3 ORF12 Deletion

(A) Fish were housed in SCT at 24°C. At time 0, fish were infected with the 12Del or 12Rev versions of CyHV-3. Cumulative survival rates of carp (n = 20) mock-infected or infected with the indicated doses of CyHV-3 ORF12 recombinant strains were measured (left graph). The right graph presents the kinetics of viral load according to time post-infection with 12Rev or 12Del (dose of 100 pfu/mL). At the indicated times post-infection, viral load was measured in gills (a total of 6 fish originating from duplicate tanks [3 fish per tank] were analyzed per time point, mean + SD). For each time point, the results obtained for 12Rev and 12Del were analyzed for significant differences (marked by asterisks).

(B) Carp (n = 15 per tank) were housed in 6 MCTs. On day 0, fish were infected with 12Rev (left column, 3 upper graphs) or 12Del (right column, 3 upper graphs). The number of fish in each compartment was expressed as a mean per day + SD. The two lower graphs represent the global analysis of the data from the three replicates as described in Figure 1B. The statistical significance of the differences between the mean number of fish observed for 12Del and 12Rev groups is presented in the right graph.

(**C**) Carp (n = 15 per tank) were housed in 6 MCTs. On day 0, fish were infected with 12Rev (3 tanks) or 12Del (3 tanks). One randomly selected tank per viral genotype was selected for monitoring the position of the fish (left graph), and the two remaining tanks were used to analyze viral load according to time post-infection. At the indicated post-infection times, viral load was measured in gills (per time point, a total of 6 fish originating from the duplicate tanks [3 fish/tank], mean + SD). For each time point, the results obtained for 12Rev and 12Del were analyzed for significant differences (marked by asterisks).

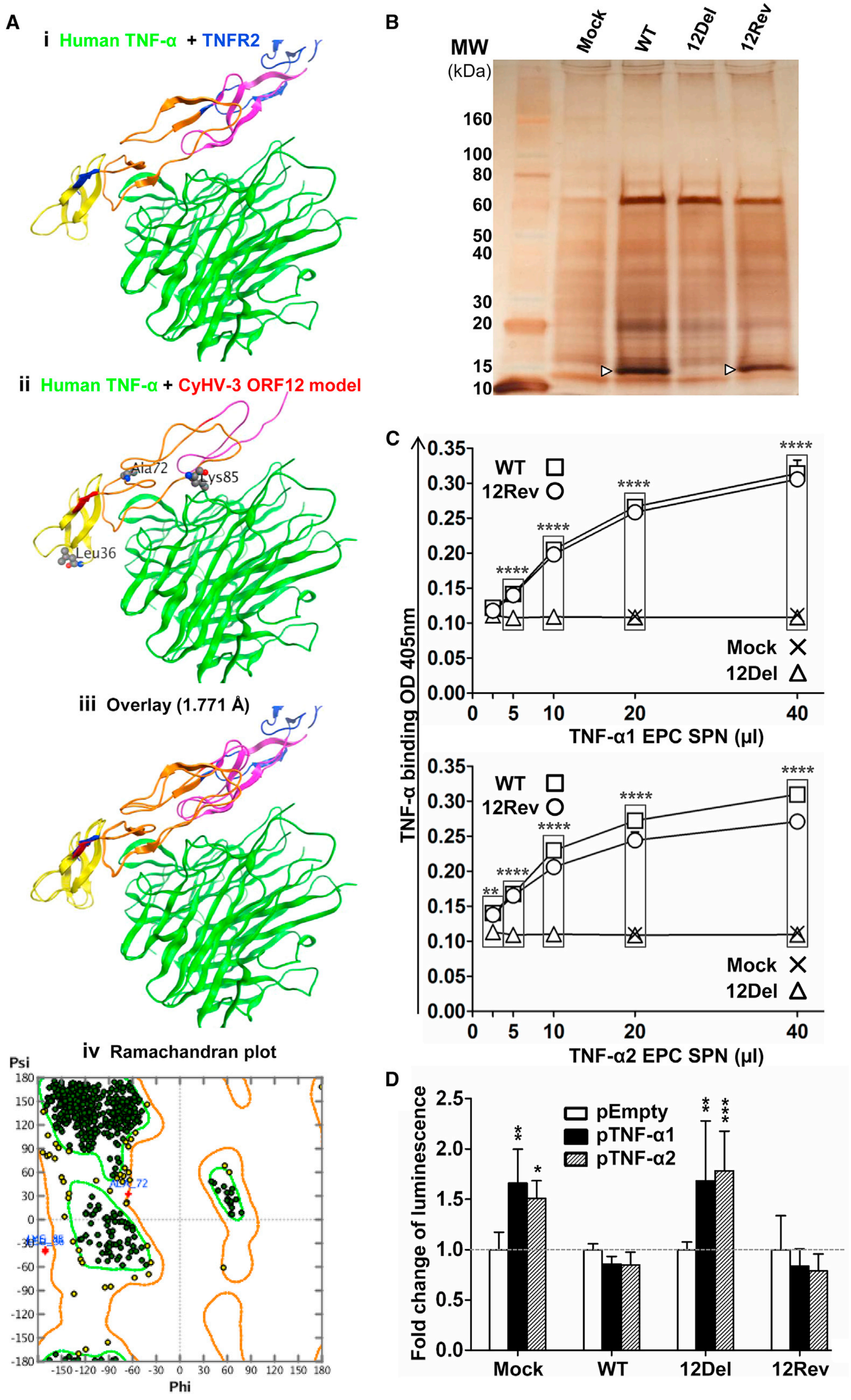

(legend on next page)

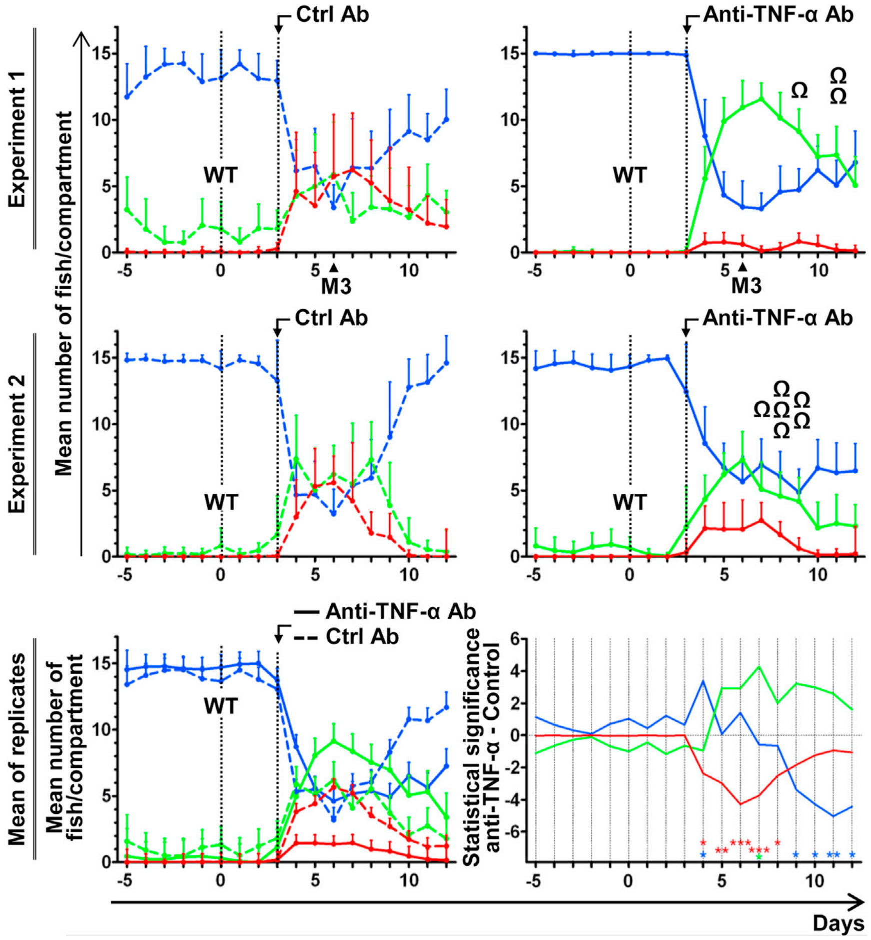

Figure 4. Anti-TNF-α Antibodies Inhibit the Expression of Behavioral Fever

Carp (n = 15 per tank) were housed in 4 MCTs. On day 0, fish were infected with wild-type CyHV-3 (WT) and then injected 3 days later with control (Ctrl) (left column, 2 upper graphs) or anti-TNF-α (right column, 2 upper graphs) antibodies. Distribution of fish in the MCT compartments was analyzed and expressed as a mean per day + SD. The Ω symbol illustrates fish that died from the infection. See also Movie S3 recorded 6 dpi for the first replicate. The two lower graphs represent the global analysis of the data from the two replicates as described in Figure 2B. The days at which the number of fish per compartment was statistically different between the control and anti-TNF-α groups are indicated according to the level of significance.

migration of fish to the 28°C compartment, suggesting the partial in vivo neutralization of TNF-α or the implication of other pyrogenic cytokines. However, these results demonstrate that carp TNF-α is a mediator of behavioral fever in our model.

DISCUSSION

Here, we studied the expression of behavioral fever by using a relevant biological model in which CyHV-3 infects its natural host through a natural route. Our data highlight the importance of the environment in the pathogen-host-environment interplay. Importantly, they also demonstrate the ability of a virus to alter the behavior of its vertebrate host through the expression of a single gene and identify the role of TNF-α as a mediator of behavioral fever in ectotherms.

Depending on the environment, CyHV-3 infection can generate extreme mortality rates. In environments that allow the expression of behavioral fever at 32°C, no mortality was observed (Figure 1C). In contrast, in some conditions in which this behavior cannot be expressed, mortality reached 100%. These observations suggest that the virulence of pathogens that infect ectotherms and are inhibited by behavioral fever can be exacerbated by environmental changes that prevent their host from expressing this innate immune response.

The present study demonstrates a beneficial effect of behavioral fever in response to a specific pathogen in a specific host. However, challenge trials using different fish species and different pathogens revealed that an increase of temperature correlated with an increase in mortality rate (exemplified by Jun et al., 2009), suggesting that expression of behavioral fever in these models would be maladaptive. It will be interesting in the future to determine whether fish can express a response against some pathogens opposite to behavioral fever (i.e., seeking cooler water) and whether this can confer an advantage to the infected subject.

Figure 3. CyHV-3 ORF12 Encodes a Soluble Decoy Receptor for TNF-α

(A) ORF12 homology modeling. (i) Human TNF-α (green) with human TNFR2 (c6 domains in yellow, orange, and pink, intervening sequences in blue; PDB: 3ALQ). (ii) Homology model (colored as in Ai) of CyHV-3 ORF12 on 3ALQ.R, with proposed interaction with TNF-α (green) also shown. Residues in ball-and-stick representation are those with poor bond angles as assessed by Ramachandran plot. (iii) Overlay of (i) and (ii), superposition at 1.771Å is achieved. (iv) Ramachandran plot. Bad angles and the position of the affected residues in (ii) are indicated in red.

(B) Silver staining of total proteins found in concentrated supernatants of CCB cells infected with CyHV-3 (WT, 12Del, or 12Rev) or mock-infected (Mock). Arrowheads indicate the band corresponding to the ORF12 protein.

(C) ELISA binding assay of carp TNF-α1 and TNF-α2 to CyHV-3 secreted proteins. The data are the mean + SD of duplicate measurements. Results for which a significant difference was observed between WT/12Rev and 12Del/Mock groups are marked by asterisks. See also Figure S2.

(D) Neutralization of carp TNF-α1 and TNF-α2 by CyHV-3 secreted proteins was tested by using a TNF-α bioluminescent reporter assay. The data are expressed as fold change of luminescence relative to the respective controls and represent the mean + SD of quadruplicate measurements. Results for which a significant difference was observed between pTNF-α1 or pTNF-α2 and pEmpty are marked by asterisks. These data are representative of three independent experiments.

Viruses are able to manipulate virtually all the physiological processes of their host to enhance their replication and transmission. However, there are very few reports of viruses that alter host behavior in a way that might increase their fitness. A frequently cited example is rabies virus, which, through the neurological lesions it causes, induces an aggressive behavior favoring the contamination of naive subjects by infectious saliva (John et al., 2015). To the best of our knowledge, there are only two reports of viruses able to alter the behavior of their host through the effect of identified genes, and both are related to baculoviruses, which have been shown to increase the locomotor activity and climbing behaviors of infected caterpillar hosts (Kamita et al., 2005; Hoover et al., 2011). To date, no pathogen has been shown to alter the expression of behavioral fever. We have demonstrated that CyHV-3 encodes a gene that delays the expression of behavioral fever, thereby enhancing viral replication in a viral excretion organ. Two non-exclusive hypotheses could explain the selective advantage conferred by the ability to delay the expression of behavioral fever. First, it could enhance viral replication and excretion by retaining infected fish at a permissive temperature. Second, it could favor viral transmission by retaining infected fish at the temperature preferred by non-infected fish, thereby promoting physical contact between them. It is notable that ORF12 delays the behavioral fever response rather than completely inhibiting it. This time-dependent effect likely reflects a selective advantage for CyHV-3. As indicated above, by delaying the behavioral fever response, ORF12 is likely to enhance viral spread through a fish population. Furthermore, by allowing fish finally to express behavioral fever, and thus survive the infection, the virus could promote the appearance of latently infected fish that will carry and spread the herpesvirus infection throughout their lives (Reed et al., 2014). Experiments are in progress to test these hypotheses.

Here, we identified the role of carp TNF-α as a key mediator of behavioral fever in an ectotherm (Figure 4). Our results suggest that behavioral fever in ectotherms and fever in endotherms are evolutionarily and functionally related through common cytokine mediators that originated more than 400 million years ago. The results of this study suggest that the ancestral signaling pathway of behavioral fever regulation in ectotherms also evolved in endotherms to regulate the expression of fever. Finally, our results support the importance of the interplay between the immune system and the central nervous system.

In conclusion, this study demonstrates the ability of a vertebrate virus to alter the behavior of its host through the expression of a single gene. This gene encodes a soluble viroreceptor able to bind and neutralize TNF-α, which was shown to be a mediator of behavioral fever in our homologous model. An interesting perspective would be to investigate whether the homology existing between the cytokine signaling pathways of behavioral fever in ectotherms and fever in endotherms extends to cytokines other than TNF-α.

EXPERIMENTAL PROCEDURES

Cells

Epithelioma papulosum cyprini (EPC) and *Cyprinus carpio* brain (CCB) cells were cultured as described previously (Boutier et al., 2015b; Forlenza et al., 2009).

TNF-α Eukaryotic Expression Vectors and Anti-TNF-α Antibodies

Plasmids pIRES-TNF-α1-EGFP and pIRES-TNF-α2-EGFP (hereafter referred to as pTNF-α1 and pTNF-α2, respectively) (Forlenza et al., 2009) were used. The empty vector, pIRES-EGFP (hereafter referred to as pEmpty), was used as a negative control. Affinity-purified polyclonal rabbit anti-carp TNF-α IgG neutralizing both carp TNF-α1 and TNF-α2 (anti-TNF-α) (Forlenza et al., 2009), as well as purified polyclonal rabbit irrelevant control IgG (CT), were used.

Production and Characterization of CyHV-3 ORF12 Recombinant Strains

A CyHV-3 ORF12 deletion (12Del) mutant and a revertant (12Rev) virus (in which ORF12 was restored) were derived from the parental, wild-type (WT) FL BAC clone (Figures S1A and S1B) by using BAC cloning and prokaryotic recombination technologies (Boutier et al., 2015b). The structure and transcription of the ORF12 region, as well as the full-length genome sequences, were validated in all viral strains (Supplemental Information and Figure S1). The ability of the recombinants to replicate in cell culture was investigated by multi-step growth curves as described previously (Boutier et al., 2015b) (Figure S1E). A more detailed version of this section is provided in the Supplemental Experimental Procedures.

Concentrated Cell Supernatants

Cultures of CCB cells were infected with CyHV-3 at a multiplicity of infection (MOI) of 0.1 plaque-forming units (pfu)/cell or mock-infected. Concentrated cell supernatants were produced as described previously (Ouyang et al., 2013).

TNF-α Binding Assay

Binding of carp TNF-α to the CyHV-3 secretome was analyzed by ELISA (Forlenza et al., 2009). A detailed description of this method is available in the Supplemental Experimental Procedures.

TNF-α Bioluminescent Reporter Assay

EPC cells stably transfected with pNiFty2-Luc (InvivoGen) (Piazzon et al., 2015), referred to as EPC-NFκB-Luc cells, were used to measure TNF-α bioactivity by bioluminescence (see the Supplemental Experimental Procedures for a detailed description of this method).

Ethics Statement

The animal studies were approved by the local ethics committee (Laboratory accreditation No. 1610008, protocol No. 1327).

Fish

European common carp (*Cyprinus carpio carpio*) between 7 and 11 months old and weighing between 8 and 12 g were used. Experimental replicates were performed with contemporary offspring derived from one female and one male carp.

Tank Systems

A single-chamber tank (SCT) system and a multi-chamber tank (MCT) system were used (Figure 1A; see also Movie S1 and Supplemental Experimental Procedures). Temperatures in all three compartments of MCTs were controlled by measurements every 30 min. The observed temperatures of the MCTs used for the experiments in this manuscript are presented as the mean ± SD in Table S1.

Inoculation and Injection of Fish

For inoculation with CyHV-3, fish were immersed for 2 hr in water containing the virus at a dose of 100 pfu/mL, unless otherwise specified. Antibodies (1 μg/g of fish body weight) were injected intraperitoneally.

Monitoring of Fish Position in the MCTs

The number of fish in each compartment was counted manually from the images captured by a digital camera at each successive 30 min, resulting in 48 measurements per day. The results are presented as the mean + SD (n = 48) of the number of fish observed per day in each compartment (for additional information see Supplemental Experimental Procedures).

Sampling of Fish Tissues

Fish were euthanized by immersion in water containing benzocaine (100 mg/L). Tissue samples were collected immediately, placed in RNAlater (Invitrogen), and stored at −80°C.

Quantification of Viral Genome Copies by qPCR

The viral genome (viral genome copies/10^6 carp *glucokinase* gene copies (log10)) was quantified by real-time TaqMan qPCR as described previously (Boutier et al., 2015b). The primers and probes are listed in the Supplemental Experimental Procedures.

Quantification of Carp Gene Expression by qRT-PCR

The expression of target genes was measured by qRT-PCR as described previously (Ouyang et al., 2013; Rakus et al., 2012). The primers used are listed in the Supplemental Experimental Procedures.

Homology Modeling

Homology models for ORF12 were constructed against 3ALQ using MOE 2015.10 (Chemical Computing Group, Montreal). The datasets for the homology models for CyHV-3 ORF12 reported in this paper are available at the Lancaster University database: http://dx.doi.org/10.17635/lancaster/researchdata/28.

Statistical Analyses

All analyses were performed by using SAS (v9.3). A detailed description of the statistical analyses used is available in the Supplemental Experimental Procedures. For all analyses, significance is set at the 0.05 threshold (–, not significant; *$p < 0.05$; **$p < 0.01$; ***$p < 0.001$; ****$p < 0.0001$).

SUPPLEMENTAL INFORMATION

Supplemental Information includes Supplemental Experimental Procedures, two figures, one table, and three movies and can be found with this article online at http://dx.doi.org/10.1016/j.chom.2017.01.010.

AUTHOR CONTRIBUTIONS

K.R., M.R., and A.V. conceived the study, performed most of the experiments, and wrote the manuscript. A.V. made the preliminary observation at the origin of this project and obtained funding for its completion. K.R. built the MCT and demonstrated the expression of behavioral fever. M.B. and J.J.-R. contributed to the experiments presented in Figures S1, S2, and 2, respectively. M.F., M.C.P., and G.F.W. designed and performed the experiments presented in Figures S2, 3C, and 3D. They also provided the antibodies used in Figure 4. A.A. and D.G. performed the in silico analyses (Figure 3A). F.F. performed statistical analyses. T.M. designed the experiments presented in Figures 2A and 2C. A.J.D. determined the genome sequences of CyHV-3 recombinants. P.B. contributed to the conceptualization of the study.

ACKNOWLEDGMENTS

We thank A. Alcami, F. Bureau, A. Chariot, and V. Mulero for discussions, C. Becco for his help in the setting of the automated picture recording system, L. Leinartz for video editing, J. Collard for graphic editing, and N. Suárez for generating genome sequence data. This work was supported by the University of Liège (Post-doc IN program), the Belgian Science Policy (BELVIR IAP7/45), the "Fonds National Belge de la Recherche Scientifique" (T.0153.13 and 1.5.176.12), an Investigator FCT development grant (IF/00641/2013), the European Community's Seventh Framework Programme (FP7/2007-2013) under Grant agreement PIEF-GA-2011-302444 FISHIL10 (M.C.P.), the Netherlands Organization for Scientific Research (NWO) under Veni project number 11200 (M.F.), and the UK Medical Research Council (MC_UU_12014/3 to A.J.D.).

Received: October 4, 2016
Revised: December 19, 2016
Accepted: January 21, 2017
Published: February 8, 2017

REFERENCES

Boehrer, B., and Schultze, M. (2008). Stratification of lakes. Reviews of Geophysics *46*, 1–27.

Boutier, M., Ronsmans, M., Rakus, K., Jazowiecka-Rakus, J., Vancsok, C., Morvan, L., Peñaranda, M.M.D., Stone, D.M., Way, K., van Beurden, S.J., et al. (2015a). Chapter Three - Cyprinid Herpesvirus 3: An Archetype of Fish Alloherpesviruses. In Adv. Virus Res, K.M. Margaret Kielian and C.M. Thomas, eds. (Academic Press), pp. 161–256.

Boutier, M., Ronsmans, M., Ouyang, P., Fournier, G., Reschner, A., Rakus, K., Wilkie, G.S., Farnir, F., Bayrou, C., Lieffrig, F., et al. (2015b). Rational development of an attenuated recombinant cyprinid herpesvirus 3 vaccine using prokaryotic mutagenesis and *in vivo* bioluminescent imaging. PLoS Pathog. *11*, e1004690.

Dinarello, C.A. (1999). Cytokines as endogenous pyrogens. J. Infect. Dis. *179* (*Suppl 2*), S294–S304.

Dinarello, C.A., Cannon, J.G., Wolff, S.M., Bernheim, H.A., Beutler, B., Cerami, A., Figari, I.S., Palladino, M.A., Jr., and O'Connor, J.V. (1986). Tumor necrosis factor (cachectin) is an endogenous pyrogen and induces production of interleukin 1. J. Exp. Med. *163*, 1433–1450.

Epperson, M.L., Lee, C.A., and Fremont, D.H. (2012). Subversion of cytokine networks by virally encoded decoy receptors. Immunol. Rev. *250*, 199–215.

Evans, S.S., Repasky, E.A., and Fisher, D.T. (2015). Fever and the thermal regulation of immunity: the immune system feels the heat. Nat. Rev. Immunol. *15*, 335–349.

Finn, R.D., Bateman, A., Clements, J., Coggill, P., Eberhardt, R.Y., Eddy, S.R., Heger, A., Hetherington, K., Holm, L., Mistry, J., et al. (2014). Pfam: the protein families database. Nucleic Acids Res. *42*, D222–D230.

Forlenza, M., Magez, S., Scharsack, J.P., Westphal, A., Savelkoul, H.F., and Wiegertjes, G.F. (2009). Receptor-mediated and lectin-like activities of carp (*Cyprinus carpio*) TNF-alpha. J. Immunol. *183*, 5319–5332.

Gilad, O., Yun, S., Adkison, M.A., Way, K., Willits, N.H., Bercovier, H., and Hedrick, R.P. (2003). Molecular comparison of isolates of an emerging fish pathogen, koi herpesvirus, and the effect of water temperature on mortality of experimentally infected koi. J. Gen. Virol. *84*, 2661–2667.

Hoover, K., Grove, M., Gardner, M., Hughes, D.P., McNeil, J., and Slavicek, J. (2011). A gene for an extended phenotype. Science *333*, 1401.

John, C.C., Carabin, H., Montano, S.M., Bangirana, P., Zunt, J.R., and Peterson, P.K. (2015). Global research priorities for infections that affect the nervous system. Nature *527*, S178–S186.

Jun, L.J., Jeong, J.B., Kim, J.H., Nam, J.H., Shin, K.W., Kim, J.K., Kang, J.C., and Jeong, H.D. (2009). Influence of temperature shifts on the onset and development of red sea bream iridoviral disease in rock bream *Oplegnathus fasciatus*. Dis. Aquat. Organ. *84*, 201–208.

Kamita, S.G., Nagasaka, K., Chua, J.W., Shimada, T., Mita, K., Kobayashi, M., Maeda, S., and Hammock, B.D. (2005). A baculovirus-encoded protein tyrosine phosphatase gene induces enhanced locomotory activity in a lepidopteran host. Proc. Natl. Acad. Sci. USA *102*, 2584–2589.

Mukai, Y., Nakamura, T., Yoshikawa, M., Yoshioka, Y., Tsunoda, S., Nakagawa, S., Yamagata, Y., and Tsutsumi, Y. (2010). Solution of the structure of the TNF-TNFR2 complex. Sci. Signal. *3*, ra83.

Netea, M.G., Kullberg, B.J., and Van der Meer, J.W.M. (2000). Circulating cytokines as mediators of fever. Clin. Infect. Dis. *31* (*Suppl 5*), S178–S184.

Ouyang, P., Rakus, K., Boutier, M., Reschner, A., Leroy, B., Ronsmans, M., Fournier, G., Scohy, S., Costes, B., Wattiez, R., and Vanderplasschen, A. (2013). The IL-10 homologue encoded by cyprinid herpesvirus 3 is essential neither for viral replication *in vitro* nor for virulence *in vivo*. Vet. Res. *44*, 53.

Piazzon, M.C., Savelkoul, H.S., Pietretti, D., Wiegertjes, G.F., and Forlenza, M. (2015). Carp Il10 has anti-inflammatory activities on phagocytes, promotes proliferation of memory T cells, and regulates B cell differentiation and antibody secretion. J. Immunol. *194*, 187–199.

Rakus, K.Ł., Irnazarow, I., Adamek, M., Palmeira, L., Kawana, Y., Hirono, I., Kondo, H., Matras, M., Steinhagen, D., Flasz, B., et al. (2012). Gene expression analysis of common carp (*Cyprinus carpio* L.) lines during Cyprinid herpesvirus 3 infection yields insights into differential immune responses. Dev. Comp. Immunol. *37*, 65–76.

Rakus, K., Ronsmans, M., and Vanderplasschen, A. (2017). Behavioral fever in ectothermic vertebrates. Dev. Comp. Immunol. *66*, 84–91.

Reed, A.N., Izume, S., Dolan, B.P., LaPatra, S., Kent, M., Dong, J., and Jin, L. (2014). Identification of B cells as a major site for cyprinid herpesvirus 3 latency. J. Virol. *88*, 9297–9309.

Yi, Y., Qi, H., Yuan, J., Wang, R., Weng, S., He, J., and Dong, C. (2015). Functional characterization of viral tumor necrosis factor receptors encoded by cyprinid herpesvirus 3 (CyHV3) genome. Fish Shellfish Immunol. *45*, 757–770.

Cell Host & Microbe, Volume *21*

Supplemental Information

Conserved Fever Pathways across Vertebrates:

A Herpesvirus Expressed Decoy TNF-α Receptor

Delays Behavioral Fever in Fish

Krzysztof Rakus, Maygane Ronsmans, Maria Forlenza, Maxime Boutier, M. Carla Piazzon, Joanna Jazowiecka-Rakus, Derek Gatherer, Alekos Athanasiadis, Frédéric Farnir, Andrew J. Davison, Pierre Boudinot, Thomas Michiels, Geert F. Wiegertjes, and Alain Vanderplasschen

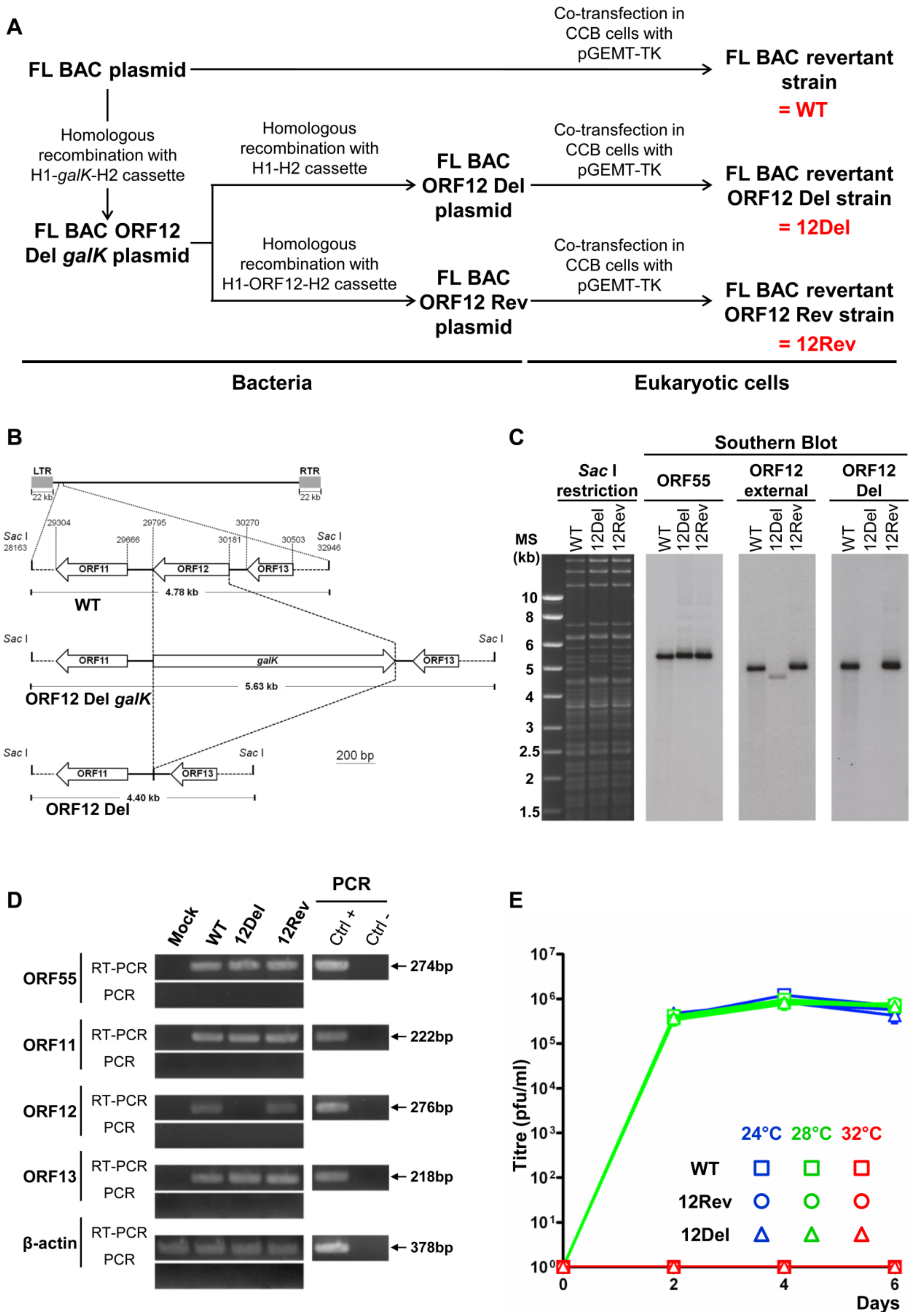

A
FL BAC plasmid
Co-transfection in CCB cells with pGEMT-TK
FL BAC revertant strain
= WT
Homologous recombination with H1-galK-H2 cassette
FL BAC ORF12 Del galK plasmid
Homologous recombination with H1-H2 cassette
FL BAC ORF12 Del plasmid
FL BAC revertant ORF12 Del strain
= 12Del
Homologous recombination with H1-ORF12-H2 cassette
FL BAC ORF12 Rev plasmid
FL BAC revertant ORF12 Rev strain
= 12Rev
Bacteria
Eukaryotic cells
B
LTR
RTR
22 kb
Sac I
28163
29304
29666
29795
30181
30270
30503
32946
ORF11
ORF12
ORF13
4.78 kb
WT
galK
5.63 kb
ORF12 Del galK
4.40 kb
ORF12 Del
200 bp
C
Southern Blot
Sac I restriction
ORF55
ORF12 external
ORF12 Del
MS (kb)
WT
12Del
12Rev
10
8
6
5
4
3
2.5
2
1.5
D
PCR
Mock
WT
12Del
12Rev
Ctrl +
Ctrl -
ORF55
ORF11
ORF12
ORF13
β-actin
RT-PCR
PCR
274bp
222bp
276bp
218bp
378bp
E
Titre (pfu/ml)
Days
24°C
28°C
32°C
WT
12Rev
12Del

Figure S1 related to Figure 2 – Production and characterization of CyHV-3 ORF12 recombinant strains. (**A**) Flow chart of steps performed to produce ORF12 recombinant plasmids and to reconstitute viral strains. (**B**) The region of the CyHV-3 genome encoding ORF12 is illustrated for wild type (WT), ORF12 Del *galK* and ORF12 Del genotypes. All coordinates correspond to the reference CyHV-3 sequence (NC_009127.1). (**C**) Structural analysis of the genome of ORF12 recombinant strains by *Sac* I restriction and Southern blotting. (**D**) RT-PCR analysis of the ORF12 genome region. (**E**) Effect of ORF12 deletion on viral growth *in vitro*. Replication kinetics of CyHV-3 ORF12 recombinant strains were compared with those of the WT strain using a multi-step growth assay at three different temperatures. The data presented are the means + SD of triplicate measurements.

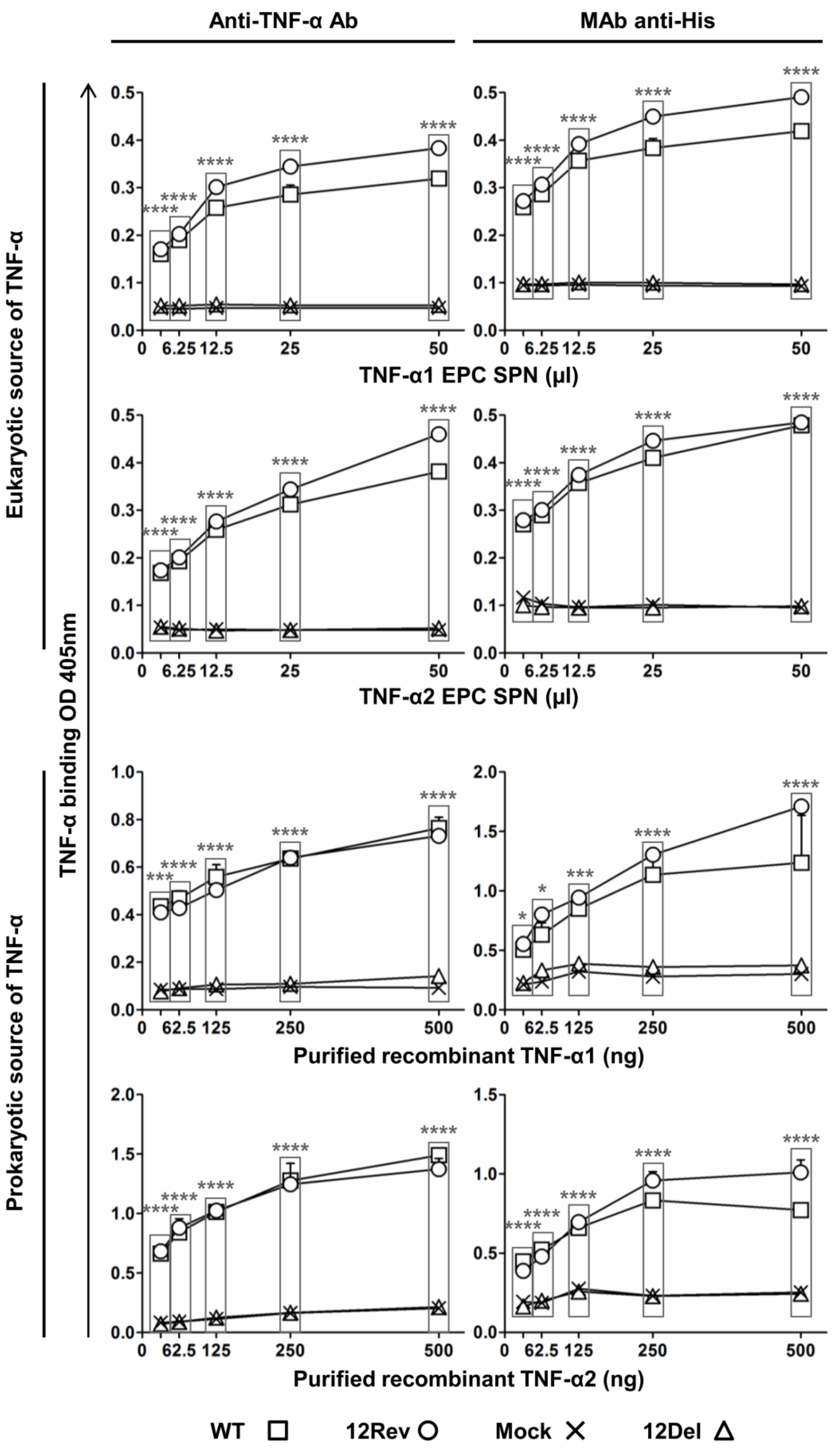

Anti-TNF-α Ab
MAb anti-His
Eukaryotic source of TNF-α
Prokaryotic source of TNF-α
TNF-α binding OD 405nm
TNF-α1 EPC SPN (µl)
TNF-α2 EPC SPN (µl)
Purified recombinant TNF-α1 (ng)
Purified recombinant TNF-α2 (ng)
WT
12Rev
Mock
12Del

Figure S2 related to Figure 3C. Binding of carp TNF-α1 and TNF-α2 to CyHV-3 secreted proteins. ELISAs were performed with two sources of carp TNF-α: supernatant of transfected EPC cells (upper half of the figure) and purified TNF-α1/ TNF-α2 expressed in bacteria (lower half). Binding of TNF-α1/ TNF-α2 was detected with polyclonal anti-TNF-α antibodies (left column) or with a MAb anti-His (right column). The data are the mean + SD of duplicate measurements. Results for which a significant difference was observed between the WT/12Rev and 12Del/Mock groups are marked by asterisks.

Movie S1 (M1) related to Figure 1B. Common carp express behavioral fever in response to CyHV-3 infection. This movie starts with an animated cartoon illustrating the structure of the MCT used in this study and how the MCTs were positioned in the room along a central axis of symmetry (warmest compartments being the closest to the center of the room). The video then presents movies of fish that were mock-infected (left and first presented MCT) or infected (right and second presented MCT) 7 days earlier with wild-type CyHV-3.

Movie S2 (M2) related to Figure 1C. Effect of water temperature on the development of CyHV-3 disease. This video starts with an animated cartoon illustrating the structure of a MCT in which the tunnels were blocked by grids. The video then presents a movie of fish that were infected 9 days earlier with wild-type CyHV-3 before their distribution into the compartments of the MCT.

Movie S3 (M3) related to Figure 4. Anti-TNF-α antibodies inhibit the expression of behavioral fever induced by CyHV-3 infection. This video starts with an animated cartoon explaining the flow chart of this experiment. Fish were first infected with CyHV-3 and then injected three days later with anti-TNF-α antibodies (left and first presented MCT) or irrelevant control antibodies (right and second presented MCT). Movies of the fish were recorded at 6 dpi.

Table S1. Temperature recorded in the compartments of MCTs (Related to Experimental Procedures (tank systems) and to the Figures listed in the first column)

Figure	Experiment	Tank	Virus/antibody	24°C compartment	28°C compartment	32°C compartment
**Fig. 1B**	**Experiment 1**	MCT	Mock	23.4 ± 0.57°C	27.9 ± 0.41°C	33.9 ± 0.42°C
		MCT	WT	22.6 ± 0.78°C	27.7 ± 0.49°C	33.9 ± 0.55°C
**Fig. 1B**	**Experiment 2**	MCT	Mock	24.8 ± 0.20°C	28.1 ± 0.14°C	32.4 ± 0.07°C
		MCT	WT	23.8 ± 0.25°C	26.8 ± 0.26°C	30.7 ± 0.51°C
**Fig. 1B**	**Experiment 3**	MCT	Mock	24.6 ± 0.23°C	29.0 ± 0.05°C	32.1 ± 0.39°C
		MCT	WT	24.9 ± 0.88°C	27.9 ± 0.80°C	31.6 ± 0.92°C
**Fig. 1C**		MCT	WT	24.6 ± 0.40°C	28.1 ± 0.14°C	32.2 ± 0.25°C
		MCT blocked	WT	22.6 ± 0.78°C	27.7 ± 0.49°C	33.9 ± 0.55°C
**Fig. 1D**	**Observation**	MCT1	WT	24.6 ± 0.32°C	28.1 ± 0.21°C	31.7 ± 0.50°C
		MCT2	WT	24.6 ± 0.30°C	28.0 ± 0.30°C	31.7 ± 0.52°C
**Fig. 1D**	**Gene expression**	MCT1	WT	24.6 ± 0.33°C	28.1 ± 0.20°C	31.7 ± 0.47°C
		MCT2	WT	24.7 ± 0.24°C	28.1 ± 0.22°C	31.6 ± 0.24°C
		MCT3	WT	25.0 ± 0.28°C	28.2 ± 0.17°C	31.2 ± 0.11°C
		MCT4	WT	24.0 ± 0.29°C	28.0 ± 0.17°C	31.7 ± 0.25°C
**Fig. 2B**	**Experiment 1**	MCT	12Rev	24.2 ± 0.33°C	28.1 ± 0.20°C	32.3 ± 0.07°C
		MCT	12Del	23.8± 0.33°C	27.8 ± 0.41°C	32.5 ± 0.32°C
**Fig. 2B**	**Experiment 2**	MCT	12Rev	24.8 ± 0.32°C	28.2 ± 0.19°C	32.1 ± 0.10°C
		MCT	12Del	24.2± 0.21°C	27.9 ± 0.20°C	32.5 ± 0.49°C
**Fig. 2B**	**Experiment 3**	MCT	12Rev	24.5 ± 0.11°C	27.4 ± 0.16°C	32.4 ± 0.24°C
		MCT	12Del	24.1± 0.16°C	27.7 ± 0.16°C	32.9 ± 0.15°C
**Fig. 2C**	**Observation**	MCT	12Rev	23.6 ± 0.16°C	27.6 ± 0.26°C	32.3 ± 0.24°C
		MCT	12Del	23.8 ± 0.35°C	28.2 ± 0.18°C	32.5 ± 0.20°C
**Fig. 2C**	**Viral load**	MCT1	12Rev	24.5 ± 0.20°C	27.9 ± 0.17°C	32.3 ± 0.38°C
		MCT2	12Rev	23.6 ± 0.35°C	28.5 ± 0.48°C	32.4 ± 0.20°C
		MCT1	12Del	24.8 ± 0.21°C	28.1 ± 0.17°C	32.2 ± 0.23°C
		MCT2	12Del	24.9 ± 0.21°C	28.1 ± 0.18°C	32.2 ± 0.22°C
**Fig. 4**	**Experiment 1**	MCT	WT/ CT	23.5 ± 0.22°C	28.2 ± 0.31°C	32.6 ± 0.10°C
		MCT	WT/ anti-TNF-α	23.8 ± 0.29°C	28.5 ± 0.24°C	33.0 ± 0.27°C
	**Experiment 2**	MCT	WT/ CT	24.4 ± 0.44°C	28.3 ± 0.43°C	32.8 ± 0.46°C
		MCT	WT/ anti-TNF-α	24.2 ± 0.48°C	28.3 ± 0.53°C	32.6 ± 0.72°C

Supplemental Experimental Procedures

Production and characterization of CyHV-3 ORF12 recombinant strains

CyHV-3 ORF12 recombinant strains were produced by using BAC cloning and prokaryotic recombination technologies (Figure S1). The parental plasmid was the CyHV-3 FL BAC clone (Boutier et al., 2015b). Recombinant plasmids were produced by using galactokinase (*galK*) positive/negative selection in bacteria (Boutier et al., 2015b) (Figure S1A) before reconstitution of infectious virus by transfection into CCB cells. The first recombination process (*galK* positive selection) replaced ORF12 by *galK*, resulting in the FL BAC ORF12 Del *galK* plasmid. Recombination was achieved by using the H1-*galK*-H2 recombination cassette, which consisted of the *galK* gene flanked by 50 bp sequences of the CyHV-3 genome flanking ORF12. This recombination cassette was produced by PCR by using the p*galK* vector as a template and the primers listed in the Table below. The second recombination process (*galK* negative selection) removed the *galK* gene (FL BAC ORF12 Del plasmid) or replaced the *galK* gene by the CyHV-3 wild type ORF12 sequence (FL BAC ORF12 Rev plasmid). The FL BAC ORF12 Del plasmid was obtained by recombination with the H1-H2 cassette. This cassette consisted of 50 bp of the CyHV-3 genome upstream and downstream of ORF12. The FL BAC ORF12 Rev plasmid was produced by recombination with the H1-ORF12-H2 cassette. This cassette was produced by PCR using the primers listed in the Table below and CyHV-3 FL DNA as the template. To reconstitute infectious virus, the recombinant BAC plasmids were co-transfected with the pGEMT-TK plasmid (molecular ratio of 1:75) in CCB cells. Transfection with the pGEMT-TK plasmid induced recombination upstream and downstream of the BAC cassette, leading to its complete removal and consequent reversion to a wild-type ORF55 locus (FL BAC revertant strains). Plaques negative for enhanced green fluorescent protein (EGFP) expression (encoded by the BAC cassette) were picked and amplified.

The structure (Figure S1C) and transcription (Figure S1D) of the ORF12 region of CyHV-3 recombinants were characterized by Southern blot and RT-PCR analyses, respectively. Probes for Southern blot analyses were produced by PCR using the primers listed in the Table below. Transcriptional analyses by RT-PCR were performed as described previously (Boutier et al., 2015b) with the primers listed in the Table below.

For genetic characterization of CyHV-3 recombinants by full length genome sequencing, DNA (500 ng) was sheared by sonication to an average size of 400 bp and prepared for sequencing by using a KAPA library preparation kit (KAPA Biosystems). The fragments were A-tailed, ligated to the NEBnext Illumina adaptor (New England Biolabs), and amplified by PCR. Index tags were added by six cycles of PCR using KAPA HiFi HotStart and NEBnext indexing primers. The libraries were analyzed by using a MiSeq DNA sequencer running v2 chemistry (Illumina). Approximately 1 million 250-nucleotide paired-end reads were obtained per sample. The reads were prepared for assembly by using Trim Galore v. 0.2.2 (http://www.bioinformatics.babraham.ac.uk/projects/trim_galore). Sequence accuracy was checked by using BWA v. 0.6.2-r126 (Li and Durbin, 2010) to assemble the reads against the sequence of the parental strain (KP343683) adapted by the conceptual mutagenesis performed and visualizing the alignment by using Tablet v. 1.13.08.05 (Milne et al., 2013). The 12Rev virus exhibited a sequence identical to the parental sequence. As intended, the 12Del virus completely lacks ORF12 (29795-30181). It also contains two nucleotide substitutions, the first a G to A transition at 240846 and the second an A to G transition at 58571. The former is a synonymous substitution in ORF139, and the second a nonsynonymous substitution in ORF36 resulting in a N to D change, a polymorphism found in CyHV-3 and in the orthologue of CyHV-2. In all viral genomes, two regions were of undetermined length: an A repeat and a GA repeat located at 32540-32465 and 177568-177730 nt, respectively, in the CyHV-3 reference sequence (NC_009127.1).

The fitness of the CyHV-3 recombinants to replicate in cell culture was investigated by multi-step growth curves as described previously (Boutier et al., 2015b). Briefly, cultures of CCB cells were inoculated in triplicate with CyHV-3 at a MOI of 0.1 pfu/cell. Supernatants were collected from the infected cultures at successive intervals and stored at -80°C. Titration of infectious viral particles was determined by duplicate plaque assays.

TNF-α binding assay

Binding of carp TNF-α to the CyHV-3 secretome was analyzed by ELISA (Forlenza et al., 2009). ELISA plates were coated with 5 µg total protein from concentrated CCB supernatants. After blocking, plates were incubated with the supernatants from EPC cells that had been transfected previously with pTNF-α1, pTNF-α2 or pEmpty, or with purified carp TNF-α1/TNF-α2 expressed in bacteria (Forlenza et al., 2009). Bound TNF-α was quantified by using two detection systems: rabbit anti-TNF-α antibodies followed by goat anti-rabbit IgG-HRP, or mouse monoclonal (MAb) anti-His antibody (Quiagen) followed by goat anti-mouse IgG-HRP (Forlenza et al., 2009).

TNF-α bioluminescent reporter assay

EPC cells stably transfected with pNiFty2-Luc (InvivoGen) (Piazzon et al., 2015), hereafter referred to as EPC-NFκB-Luc cells, were used to measure TNF-α bioactivity. Supernatants from EPC cells, which had been transfected previously with pTNF-α1, pTNF-α2 or pEmpty (Forlenza et al., 2009), were pre-incubated with concentrated supernatants of CyHV-3-infected or mock-infected CCB cells (40 µg total protein) for 30 min at RT to allow ORF12-TNF-α binding. EPC-NFκB-Luc cells seeded in 96-well plates were stimulated with 50 µl of the pre-incubated mixtures described above. After incubation for 6 h at 27°C, cells were lysed and bioluminescence was measured. The fold change of luminescence was calculated by dividing the light units obtained for each sample by the result obtained for the respective control sample.

Tank systems

A single chamber tank (SCT) system and a multi-chamber tank (MCT) system were used in this study (Figure 1A; see also Supplemental Information M1). The SCT system consisted of an 80 L single compartment tank (width x depth x height: 0.5 x 0.4 x 0.4 m) with a mean constant temperature of approximately 24, 28 or 32°C. The MCT system consisted of a 144 L tank (width x depth x height: 1.2 x 0.3 x 0.4 m) subdivided into three equal compartments by two rigid thermal insulation panels (6 cm thick and made of polyurethane foam). Neighboring compartments were connected by a 0.4 m long transparent tunnel with a square cross-section (8 x 8 cm) placed at the bottom of the tank. Each of the three chambers had independent aeration, circulation and filtering systems. A thermal gradient (24°C - 28°C - 32°C) was established between the three chambers by cooling the first compartment and by increasing heating of the second and third compartments. Temperatures in all three compartments were controlled by measurements every 30 min. The observed temperatures of the MCTs used for the experiments in this manuscript are presented as the mean ± SD in Table S1. To simplify reading of the manuscript, the theoretical gradient of 24°C - 28°C - 32°C is presented in the manuscript using the color code described in the legend of Figure 1. The MCTs were placed in the experimental room as pairs around a central axis of symmetry, with the 32°C compartments being close to this axis and the 24°C compartments being furthest away. The positions of the experimental groups were swapped systematically between replicate tanks. In the MCTs, daily feeding was performed in the 24°C compartment independently of the position of the fish.

Monitoring of fish position in the MCTs

Fish (n=15) were initially introduced into the 24°C compartment of the MCT, in which the tunnels were obstructed by grids to prevent migration out of the compartment. After an acclimatization period of 3 weeks, the grids were removed, and the distribution of fish into the three compartments was monitored over time. A digital camera (Logitech HD Webcam c310) placed in front of each MCT recorded pictures every 3 min. The number of fish in each compartment was counted manually from the images captured at each successive 30 min, resulting in 48 measurements per day. When the positions of the fish were not compatible with an accurate count, measurements were made from the previous or following image (i.e. collected 3 min before or after image examined initially). The results are presented as the mean + SD (n=48) of the number of fish observed per day in each compartment.

Statistical analyses

Most statistical analyses were performed by using linear models. The observations on fish position in MCTs (Figure 1B and D, Figure 2B and Figure 4) were analyzed by using daily averages of the number of fish present in each compartment for each replicate as follows: a mixed model was set up with these averages as dependent variables and the status (WT or Mock, Figure 1B and D; 12Rev or 12Del, Figure 2B; and Ctrl or anti-TNF-α, Figure 4), the time and the interaction between time and status as independent variables. Due to the lack of some observations (e.g. on the day of infection or at the end of the observation period or upon deaths), averages might have been computed over unequal number of observations, requiring a weighting of the data (using the corresponding number of observations) in order to estimate the standard errors correctly. Potential correlations between successive measurements over the same set of fish were taken into account by a type 1 auto-regressive structure using an Akaike Information Criterion comparison. Obtained estimators were then globally compared using type 3 tests of the fixed effects of the model with a Kenward-Roger correction for the degrees of freedom. Post-hoc comparisons of the sets of fish (WT or Mock, Figure 1B and D; 12Rev or 12Del, Figure 2B; and Ctrl or anti-TNF-α, Figure 4) were then obtained for each day of the experiment, testing the null hypothesis that the means are actually equal. Differences in viral load (data after log transformation) and cytokine expression (data after log transformation) (Figure 1D) between CyHV-3 infected fish collected at various post-infection times and mock-infected fish sampled at day 0 were analyzed by using a one-way ANOVA. Post-hoc comparisons between days were performed by using Tukey's test. These analyses were performed independently for each cytokine. The TNF-α binding assay (Figure 3C

and Figure S2), TNF-α neutralization assay (Figure 3D), virus load measurement (Figure 2A and C), and multi-step growth curves (Figure S1E) were analyzed by using two- or three-way ANOVA. More precisely, for the binding assay of TNF-α, the level of binding was modeled by using a linear model involving cell supernatants, TNF-α1 or TNF-α2 volumes as well as their interactions. For the neutralization assay of TNF-α biological activity, the fold change of luminescence (ratio of the TNF-α1 or TNF-α2 levels respective to the control supernatant level) was modeled by using a linear model involving cell supernatants, cytokine (TNF-α1, TNF-α2 or control) supernatants, and their interactions. For viral load, the viral genome copies (data after log transformation) were modeled by using a linear model involving virus strains, tanks, and their interactions. For multi-step growth curves, the logarithm of the titre was modeled by using a linear model involving the day, viral strains, temperature, and their interactions. For the survival analyses (Figure 1C and 2A), a standard SAS LIFETEST procedure was performed. Survival curves were compared pairwise. Statistical significance is represented as follows: -, not significant; *, $p<0.05$; **, $p<0.01$; ***, $p<0.001$; and ****, $p<0.0001$. P-values <0.05 were considered significant.

Primer sets used in this study (related to Supplemental Experimental Procedures)

	Primer name	Sequence (5'- 3')	Coordinates*/ accession number
**Synthesis of recombination cassettes**			
**Cassette name**			
H1-*galK*-H2	ORF12 *galK* F	AGGCTGCACTGCTGCGCACAGTGACGAGTAGACGGTGGGA GGTCGGTGAACCTGTTGACAATTAATCATCGGCA	29745-29794
	ORF12 *galK* R	CTTGTTTTACTATACTCTATCACATCTCCGACTTGATTTCTT CTCAAACCTCAGCACTGTCCTGCTCCTT	30231-30182
H1-ORF12-H2	ORF12 Fw RVT	ATGAAGAGTTTGTGTCGAGC	29668-29687
	ORF12 Rev RVT	GGCTACGTATAACTGTCATG	30312-30293
**Synthesis of probes for Southern blot analysis**			
**Probe name**			
CyHV-3 ORF55	ORF55InF	AGCGCTACACCGAAGAGTCC	95990-96009
	ORF55stopR	TCACAGGATAGATATGTTACAAG	96516-96494
CyHV-3 ORF12 Del	ORF12 Int Fw 5'	TCGTAGTCGCCCTGACATCC	29847-29866
	ORF12 Int Rev 3'	AACTAGACACTCATCATGCGG	30122-30102
CyHV-3 ORF12 external	ORF12 Fw 5'	GAATTTATATGCAGCGAGTG	29723-29742
	ORF12 Rev 3'	TATTGTCTGTTTCTGTGCTC	30261-30242
**Transcriptional analysis**			
**Gene amplified**			
CyHV-3 ORF11	ORF11 Fw	CAACCCACAACAGCAGTACC	29595-29576
	ORF11 Rev	TTGCCCTGTCTCATCTTGGT	29374-29393
CyHV-3 ORF12	ORF12 Int Fw 5'	TCGTAGTCGCCCTGACATCC	29847-29866
	ORF12 Int Rev 3'	AACTAGACACTCATCATGCGG	30122-30102
CyHV-3 ORF13	ORF13 Fw	TGTGAGTCATGACAGTTATACGT	30286-30308
	ORF13 Rev	ATGACTGACTGGACATCGGC	30503-30484
CyHV-3 ORF55	ORF55 ATG Fw	ATGGCTATGCTGGAACTGG	95866-95884
	ORF55 In Rev	GGCGCACCCAGTAGATTATG	96467-96448
Carp *β-actin*	Actin-Fw	ATGTACGTTGCCATCCAGGC	M24113
	Actin-Rev	GCACCTGAACCTCTCATTGC	
**qPCR analysis for quantification of viral load**			
**Gene amplified**			
CyHV-3 ORF89	KHV-86F	GACGCCGGAGACCTTGTG	AF411803
	KHV-163R	CGGGTTCTTATTTTTGTCCTTGTT	
	KHV-109P	(6FAM) CTTCCTCTGCTCGGCGAGCACG (BHQ1)	
Carp *glucokinase*	CgGluc-162F	ACTGCGAGTGGAGACACATGAT	AF053332
	CgGluc-230R	TCAGGTGTGGAGCGGACAT	
	CgGluc-185P	(6FAM) AAGCCAGTGTCAAAATGCTGCCCACT (BHQ1)	
**RT-qPCR analysis for quantification of cytokine expression**			
**Gene amplified**			
Carp *40S*	40S-F	CCGTGGGTGACATCGTTACA	AB012087
	40S-R	TCAGGACATTGAACCTCACTGTCT	
Carp *il1β*	IL-1β-F	AAGGAGGCCAGTGGCTCTGT	AJ245635
	IL-1β-R	CCTGAAGAAGAGGAGGCTGTCA	
Carp *TNF-α1* and *TNF-α2*	TNF-α1 and 2-F	GCTGTCTGCTTCACGCTCAA	AJ311800 and AJ311801
	TNF-α1 and 2-R	CCTTGGAAGTGACATTTGCTTTT	
Carp *il6a*	IL-6a-F	CAGATAGCGGACGGAGGGGC	KC858890
	IL-6a-R	GCGGGTCTCTTCGTGTCTT	
Carp *il6b*	IL-6b-F	GGCGTATGAAGGAGCGAAGA	KC858889
	IL-6b-R	ATCTGACCGATAGAGGAGCG	

*Coordinates based on the reference CyHV-3 genome (Accession number: NC_009127.1)
Underlined: 50 bp corresponding to the CyHV-3 sequence. Red: sequence corresponding to *galK*

Supplemental References

Li, H., and Durbin, R. (2010). Fast and accurate long-read alignment with Burrows-Wheeler transform. Bioinformatics *26*, 589-595.

Milne, I., Stephen, G., Bayer, M., Cock, P.J., Pritchard, L., Cardle, L., Shaw, P.D., and Marshall, D. (2013). Using Tablet for visual exploration of second-generation sequencing data. Brief. Bioinform. *14*, 193-202.
